# Supplementary material for: Dynamics of recombination, X inactivation and centromere proteins during stick insect spermatogenesis
Source: PLoS Genet. 2025 Aug 18;21(8):e1011827. doi: 10.1371/journal.pgen.1011827 (PMC12393702; doi:10.1371/journal.pgen.1011827)
Supplement: S1 Text — Fig A: γH2AX distribution at various stages of spermatogenesis for three additional Timema species, acquired through the projection of stack images. Focal cells at the zygotene stage are labeled with the letter Z, cells at the pachytene stage with the letter P, elongated spermatid cells with the letters ES, while yellow arrows indicate the X chromosome. Note that at the zygotene stage, the γH2AX signal is observed at a polarized region of the nucleus, whereas at the pachytene stage, the X chromosome in T. petita displays γH2AX patches as in T. californicum, but no signal was detected in T. cristinae and T. chumash. A close-up view of the X chromosome is also shown within dashed squares for each species. Scale bar: 10 μm. Fig B: Rad51 distribution at various stages of spermatogenesis for three additional Timema species, acquired through the projection of stack images. Focal cells at the zygotene stage are labeled with the letter Z, cells at the pachytene stage with the letter P, cells in metaphase I with the letters MI, and elongated spermatid cells with the letters ES. Inset zooms on the X chromosome in pachytene are shown for T. petita, while in T. chumash, dashed circles indicate the position of the sex (X) chromosome and the associated Rad51 signal along its SMC3 axis in pachytene. Scale bar: 10 μm. Fig C: Characterization of spermatogenetic stages and X chromosome identification. (A-f) illustrate SMC3 and DAPI stainings throughout T. californicum spermatogenesis. While forming thin filaments along chromosomal axes at the onset of meiosis, SMC3 filaments appear thicker as synapsis progresses between homologs (A-J). At metaphase I, SMC3 forms cross-shaped or ring-shaped signals corresponding to rod and ring bivalents, respectively (O-P). As homologs segregate during anaphase I, SMC3 concentrates into foci near centromeres until metaphase II (Q-T). SMC3 is then lost from anaphase II onwards (U-f). Note that during the round spermatid stage, nullo-X and X-containing [file pgen.1011827.s001.docx]

**
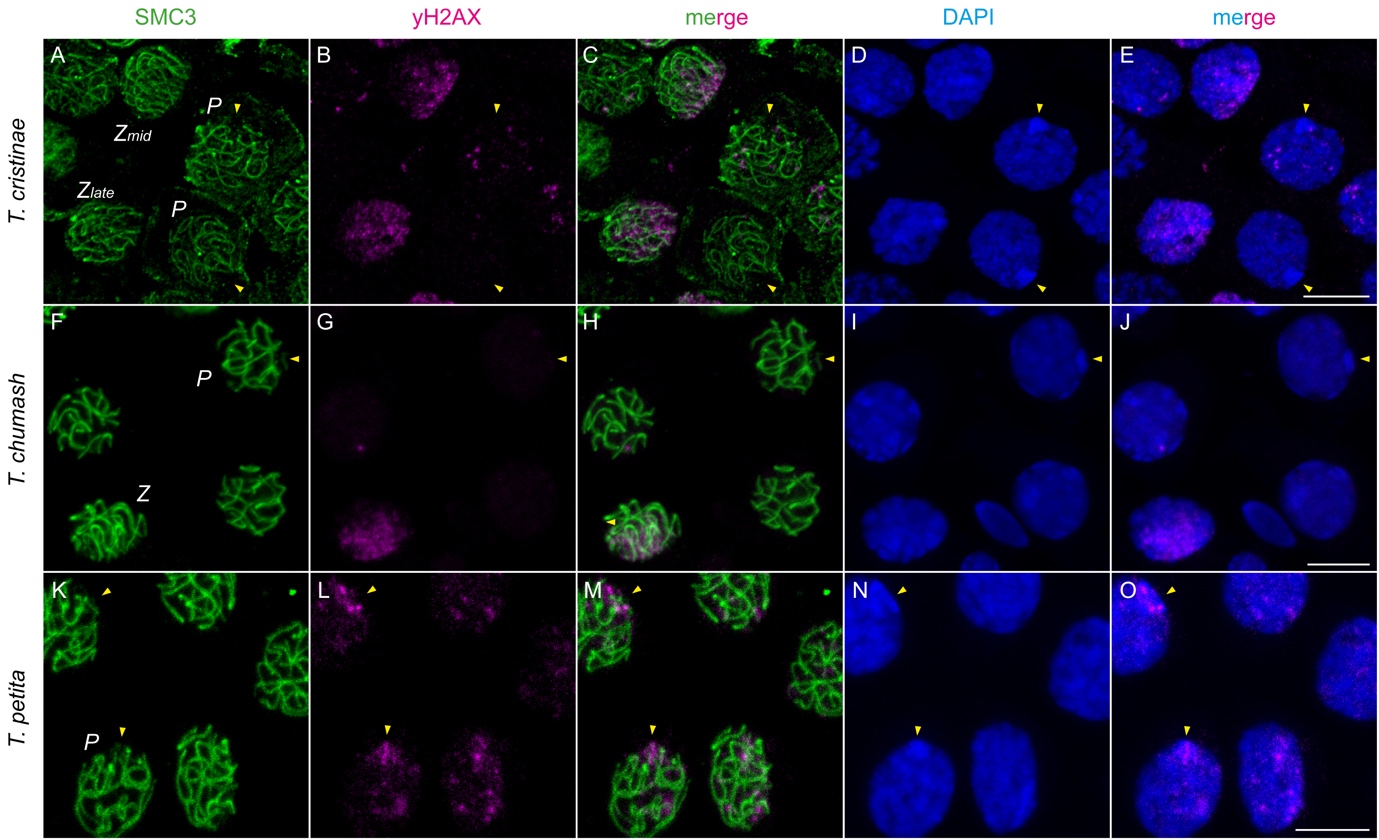
**

**Fig A:** γH2AX distribution at various stages of spermatogenesis for three additional *Timema* species, acquired through the projection of stack images. Focal cells at the zygotene stage are labeled with the letter *Z*, cells at the pachytene stage with the letter *P*, elongated spermatid cells with the letters ES, while yellow arrows indicate the X chromosome. Note that at the zygotene stage, the γH2AX signal is observed at a polarized region of the nucleus, whereas at the pachytene stage, the X chromosome in *T. petita* displays γH2AX patches as in *T. californicum*, but no signal was detected in *T. cristinae* and *T. chumash*. A close-up view of the X chromosome is also shown within dashed squares for each species. Scale bar: 10 μm.


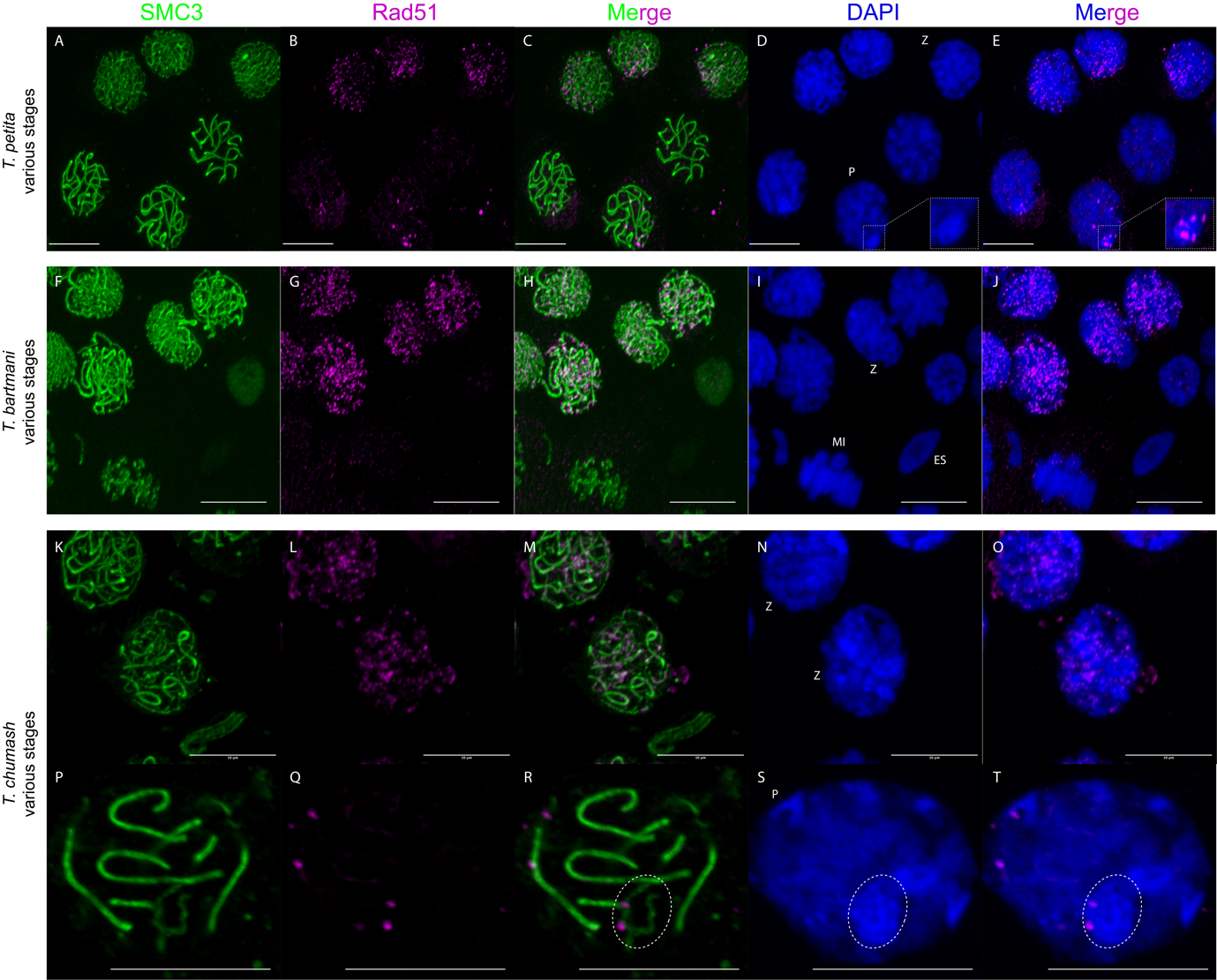


**Fig B:** Rad51 distribution at various stages of spermatogenesis for three additional *Timema* species, acquired through the projection of stack images. Focal cells at the zygotene stage are labeled with the letter *Z*, cells at the pachytene stage with the letter *P*, cells in metaphase I with the letters MI, and elongated spermatid cells with the letters ES. Inset zooms on the X chromosome in pachytene are shown for *T. petita*, while in *T. chumash*, dashed circles indicate the position of the sex (X) chromosome and the associated Rad51 signal along its SMC3 axis in pachytene. Scale bar: 10 μm.


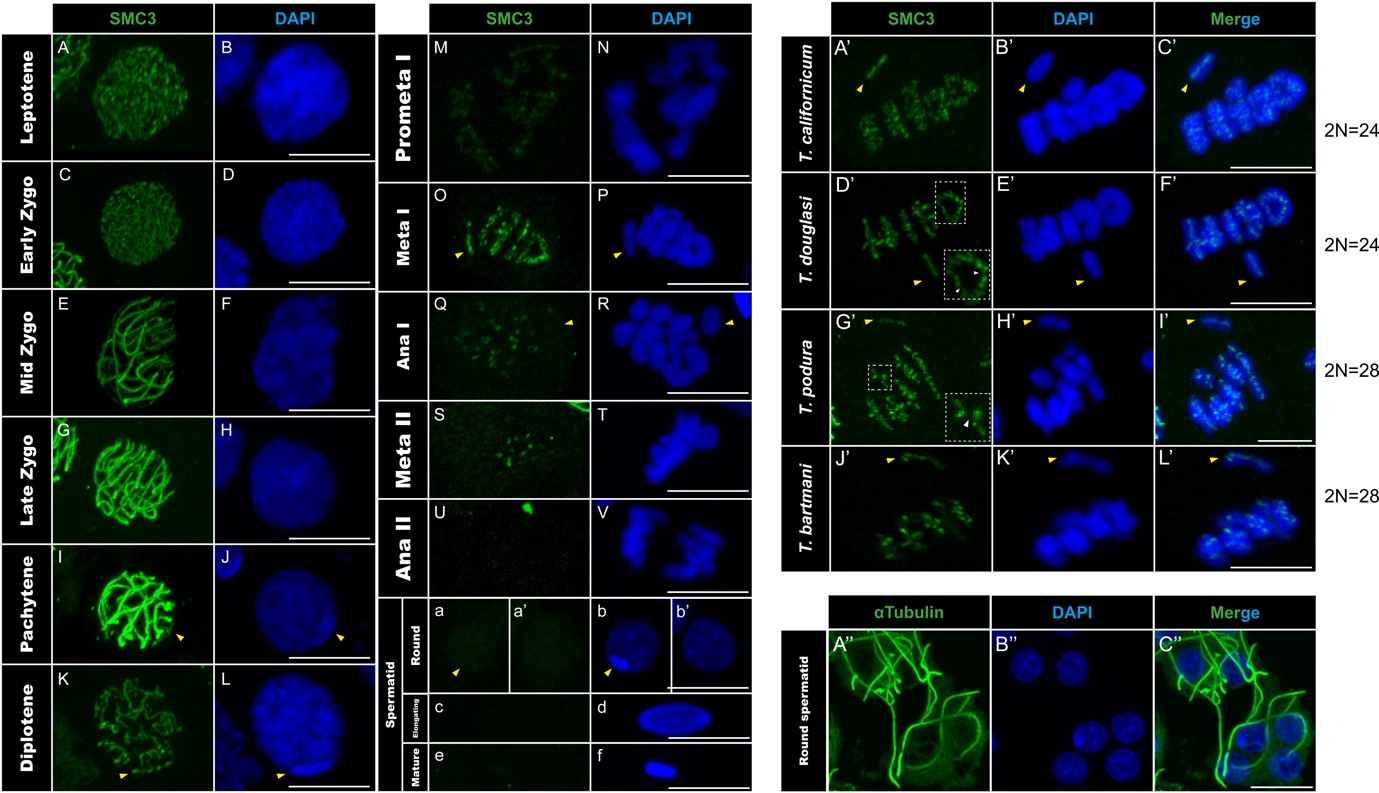


**Fig C: Characterization of spermatogenetic stages and X chromosome identification.** (A-f) illustrate SMC3 and DAPI stainings throughout *T. californicum* spermatogenesis. While forming thin filaments along chromosomal axes at the onset of meiosis, SMC3 filaments appear thicker as synapsis progresses between homologs (A-J). At metaphase I, SMC3 forms cross-shaped or ring-shaped signals corresponding to rod and ring bivalents, respectively (O-P). As homologs segregate during anaphase I, SMC3 concentrates into foci near centromeres until metaphase II (Q-T). SMC3 is then lost from anaphase II onwards (U-f). Note that during the round spermatid stage, nullo-X and X-containing spermatids are represented (a-b’). (A’-L’) illustrate rod and ring bivalents across 4 *Timema* species at metaphase I. Dashed squares indicate zooms of individual chromosomes illustrating rod and ring bivalents with interrupted SMC3 signal near chromosomal ends (white arrowheads in D’ and G’). Note that across many metaphases I analyzed, we observed consistent formation of a single, large “ring” bivalent in *T. californicum* and *T. douglasi* but not in *T. podura* and *T. bartmani*. (A’’-C’’) illustrate the formation of the flagellum during spermiogenesis as evidenced by α-tubulin staining in round spermatids. The position of the X chromosome is indicated with yellow arrowheads. Scale bar: 10 μm.


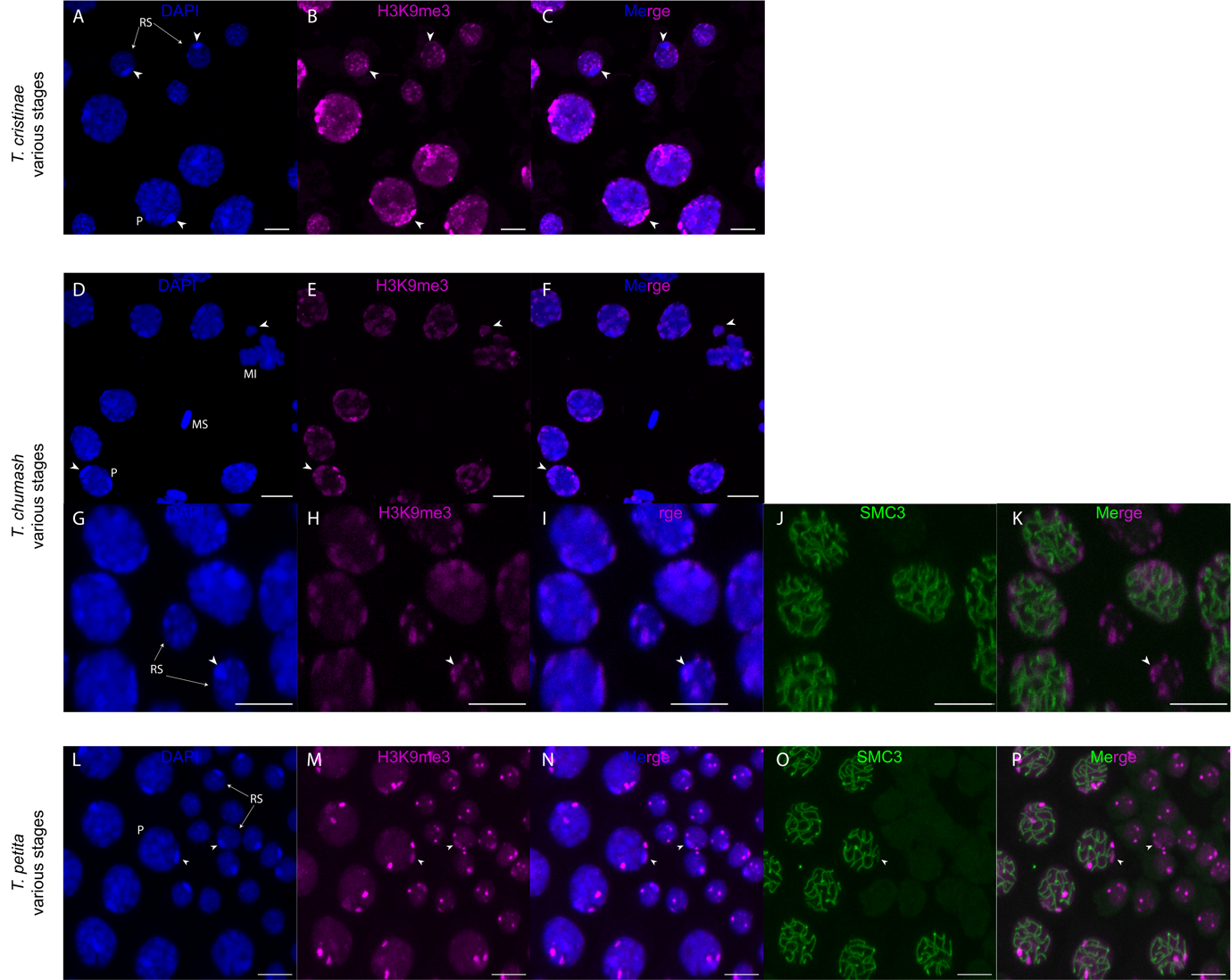


**Fig D:** H3K9me3 distribution at various stages of spermatogenesis for three additional *Timema* species, acquired through the projection of stack images. Focal cells at the pachytene stage are labeled with the letter *P*, cells in metaphase I with the letters MI, round spermatid cells with the letters RS, and mature sperm cells with the letters MS. Note the absence of SMC3 in round spermatids and the presence of H3K9me3 coating the X chromosome in *T. petita* and *T. chumash* but not in *T. cristinae*. Arrowheads indicate the location of the X chromosome. Scale bar: 10 μm.


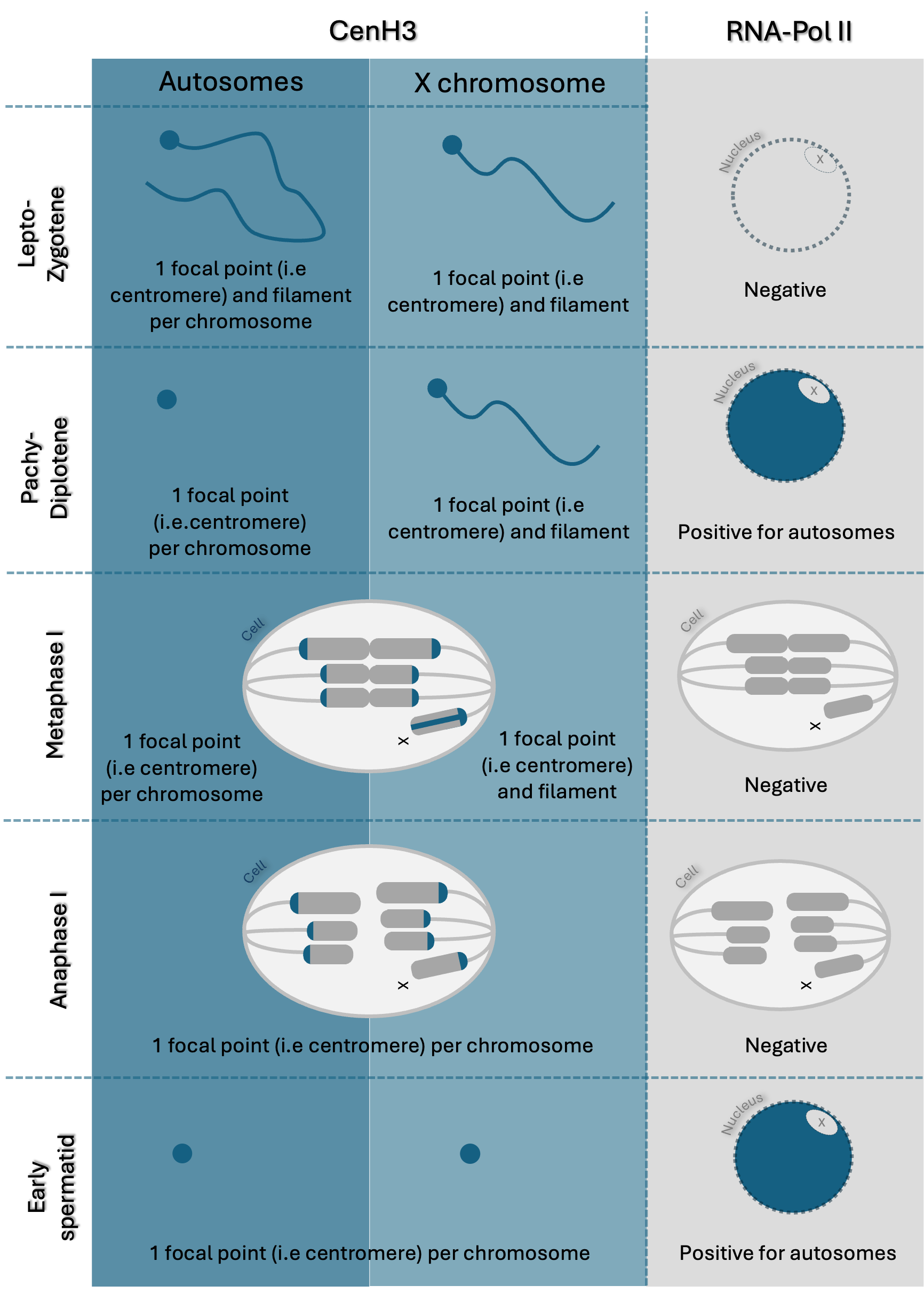


**Fig E**: Schematic representation of CenH3 and RNApol-II distributions across different stages of spermatogenesis in *Timema*. The first activation of transcription in autosomes coinsides with the shift from a longitudinal to a focal distribution of CenH3 along these same chromosomes. However, the shift of CenH3 along the X chromosome is not associated with its transcriptional activation in anaphase I. Scale bar: 10 μm.


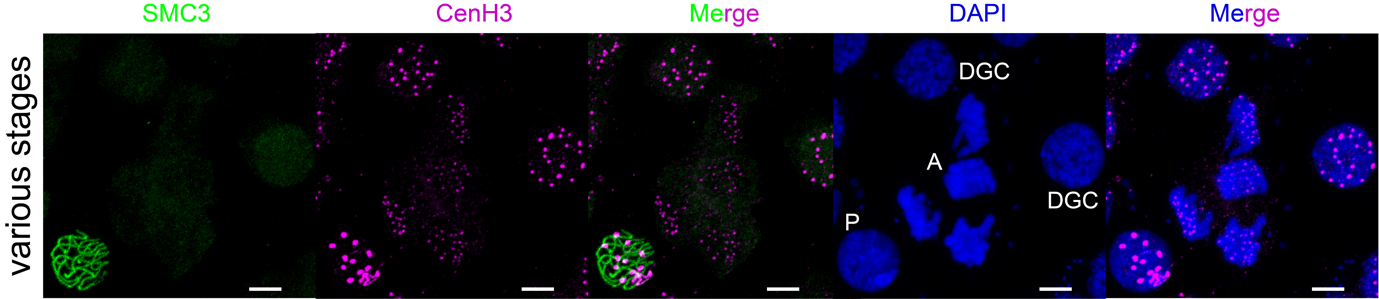


**Fig F:** CenH3 distribution in germ cells of *T. californicum* male gonads, stained with DAPI (blue) and double immunolabeled for SMC3 (green) and CenH3 (magenta). The acquisition contrasts the distribution of CenH3 for cells in meiosis (“P” for Pachytene) and before entering meiosis (“DGC” for Diploid Germ Cell and “A” for Anaphase). Scale bar: 5 μm.


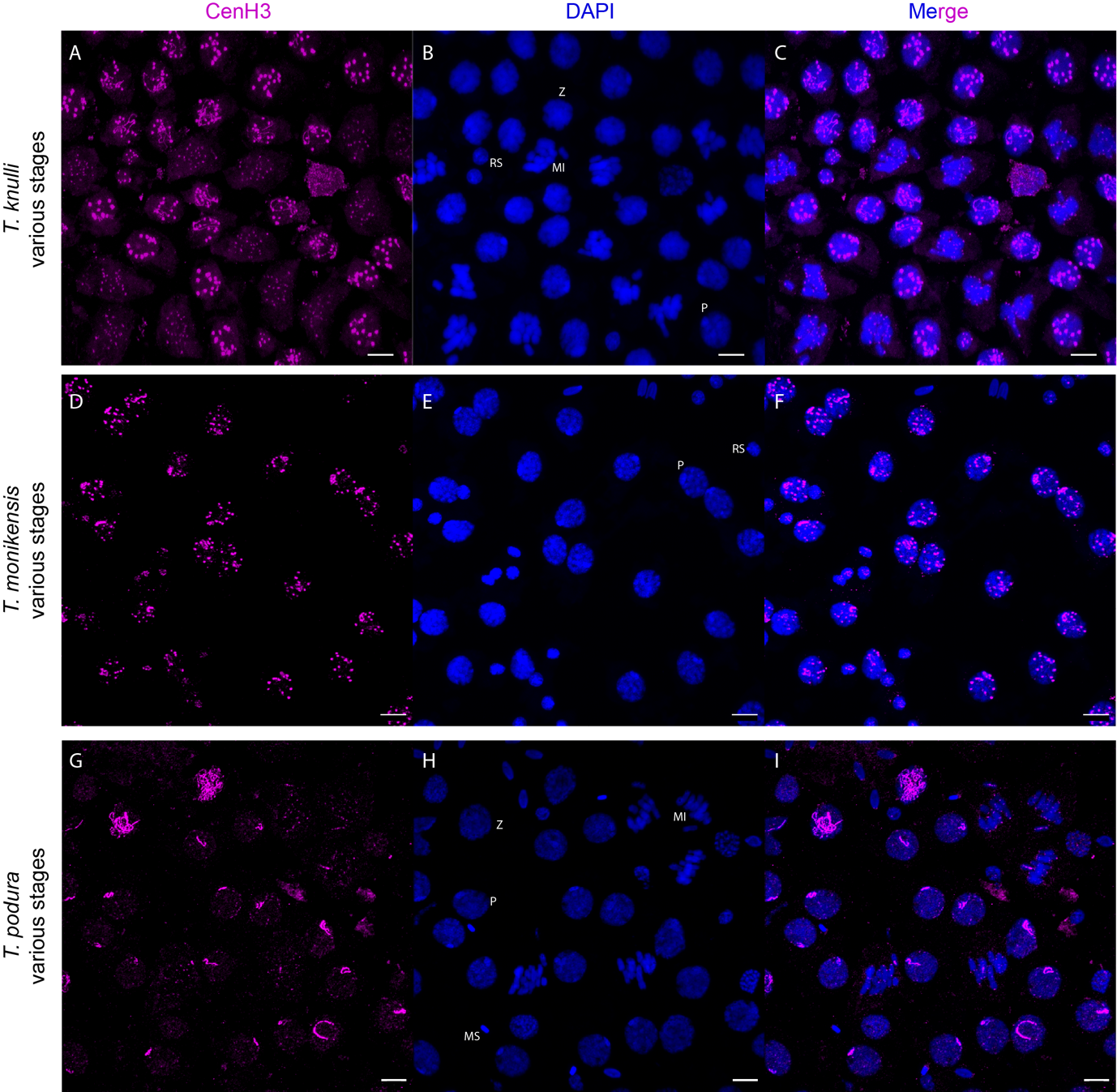


**Fig G:** Projections of stack images acquired across various stages of spermatogenesis for three additional species, stained with DAPI (blue) and immunolabeled for CenH3 (magenta). Focal cells in zygotene are labeled with letter Z, cells at the pachytene stage with the letter *P*, cells in metaphase I with the letters MI, round spermatid cells with the letters RS, and mature sperm cells with the letters MS. Scale bar: 10 μm.


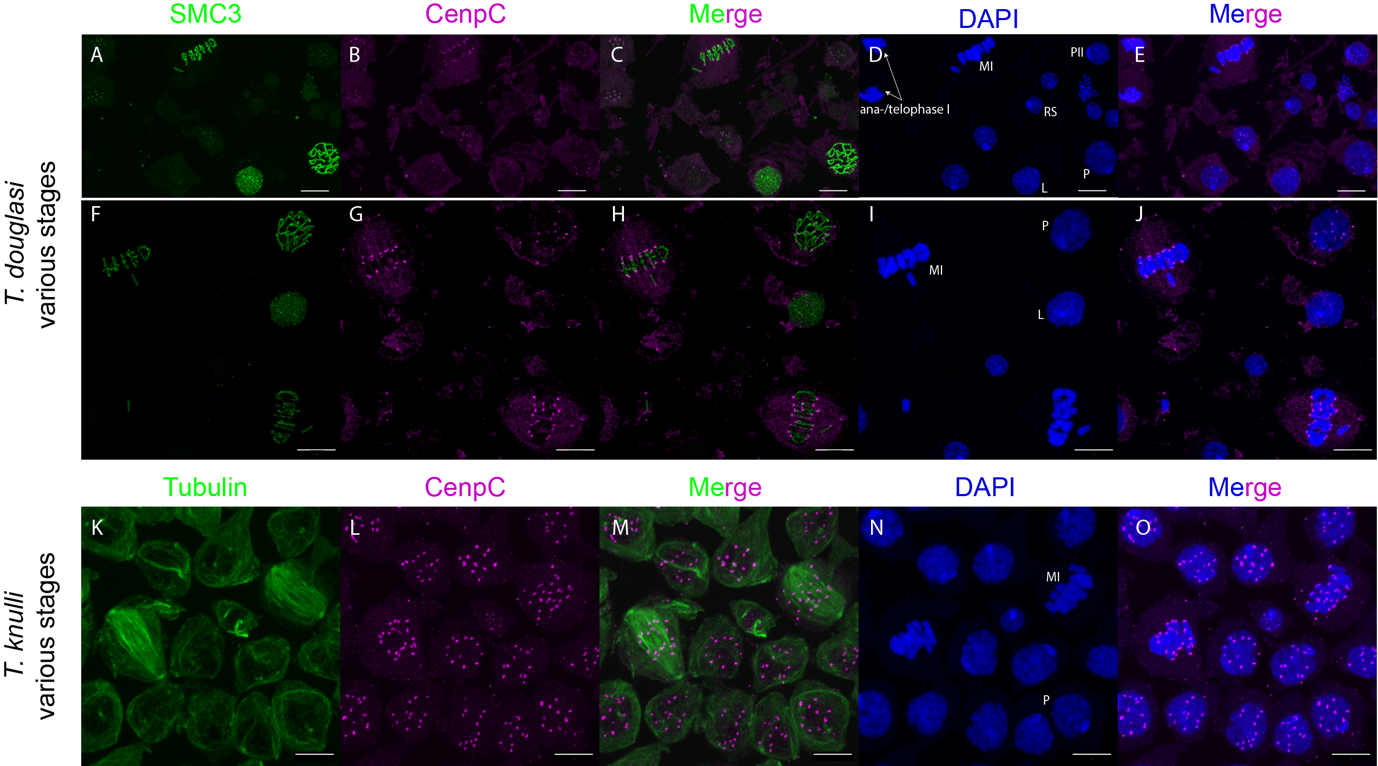


**Fig H:** CenpC distribution at various stages of spermatogenesis for two additional species, acquired through the projection of stack images. Focal cells in leptotene are labeled with the letter L, cells at the pachytene stage with the letter *P*, cells in metaphase I with the letters MI, cells in prophase II with the letters PII, and round spermatid cells with the letters RS. Note that the lower signal intensity in the upper panel is caused by a different objective and does not reflect species variation. Scale bar: 10 μm.


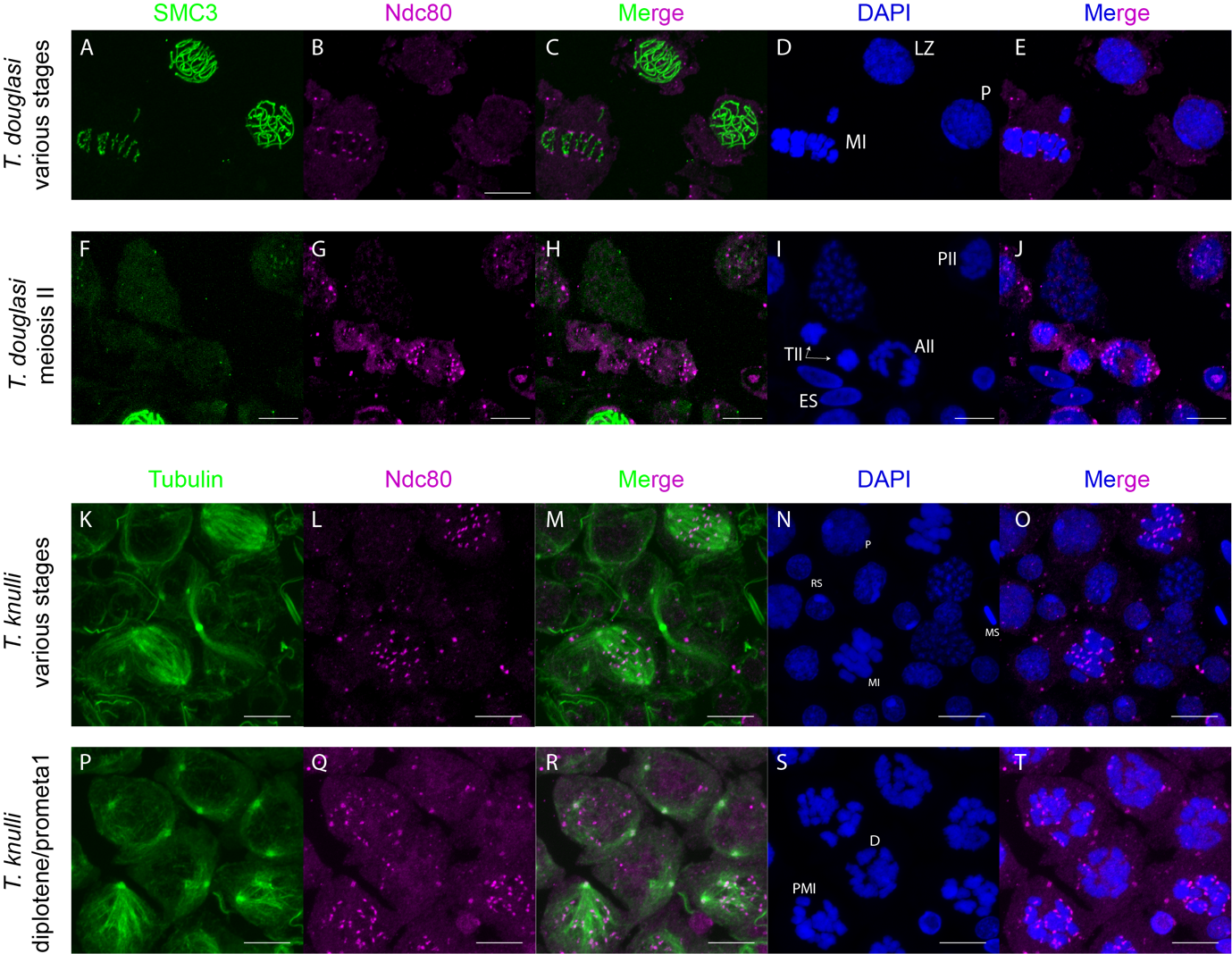


**Fig I:** Ndc80 distribution in various stages of spermatogenesis for two additional species, acquired through the projection of stack images. Focal cells at the pachytene stage are labeled with the letter *P*, cells in diplotene with the letter D, cells in pro-metaphase I with letters PMI, cells in metaphase I with the letters MI, cells in prophase II with the letters PII, cells in anaphase II with letters AII, cells in telophase II with letters TII, round spermatid cells with the letters RS, elongated spermatid cells with letters ES, and mature sperm cells with letters RS. Note that at the diplotene stage, chromosomes are already condensed but spindle microtubules (immunolabeled with 𝛂-tubulin) are not yet polymerized as compared to the pro-metaphase I stage. Scale bar: 10 μm.


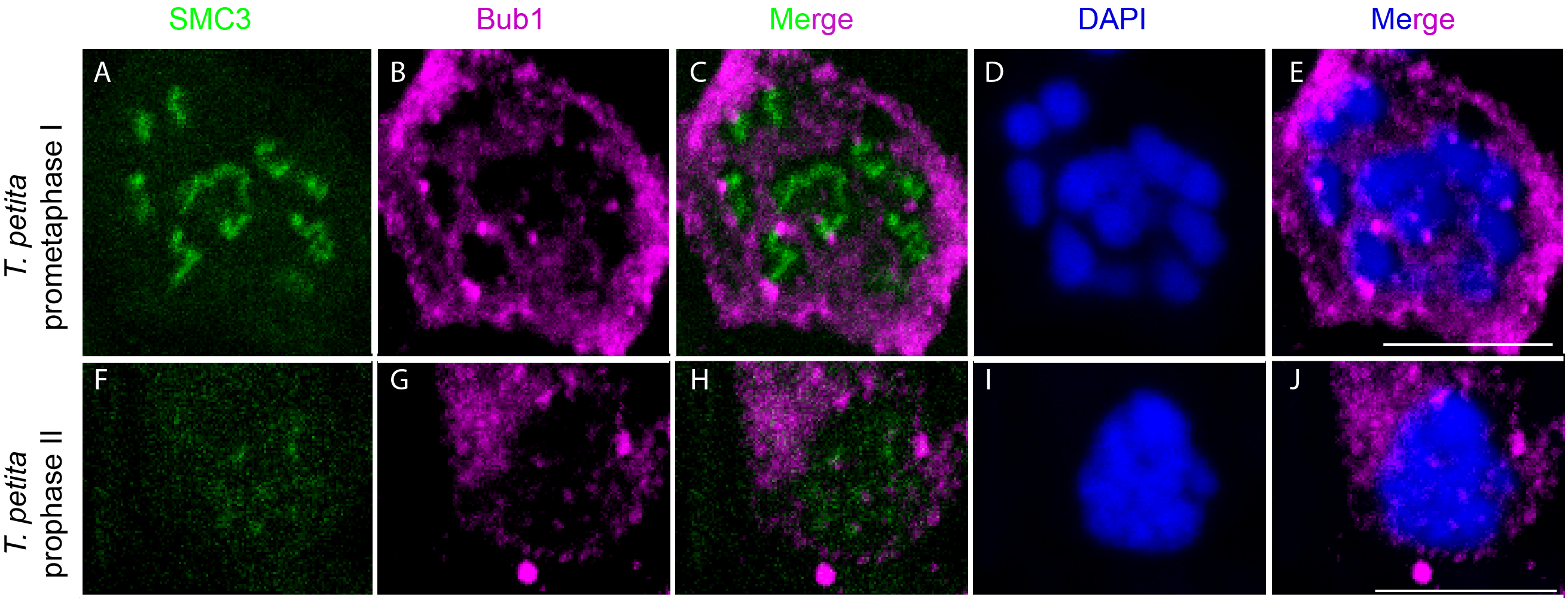


**Fig J:** Bub1 distribution at prometaphase I and prophase II stages in *T. petita*, acquired through the projection of stack images. Scale bar: 10 μm.


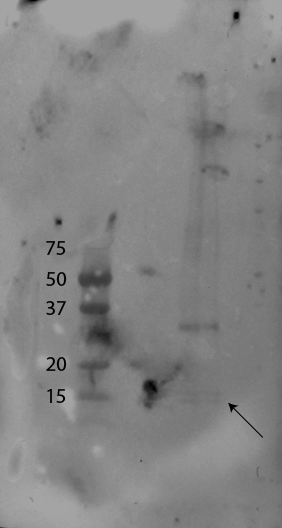


**Fig K:** Western blot of protein extracts from *Timema* testes, using the antibody against γH2AX. Molecular weight markers are indicated by numbers in kilodaltons (left) and their position by lines. The γH2AX antibody is expected to recognize a band around 15 kDa as indicted by the arrow.


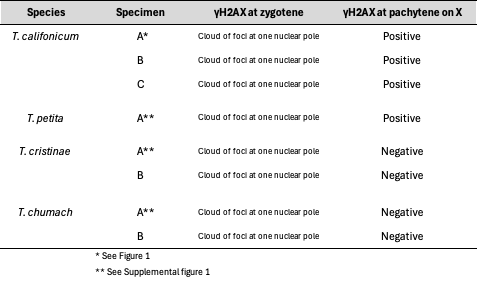


**Table A:** γH2AX distribution across species and individuals during the zygotene (bouquet) stage as well as on the X chromosome during pachytene (i.e., the initiation of MSCI).


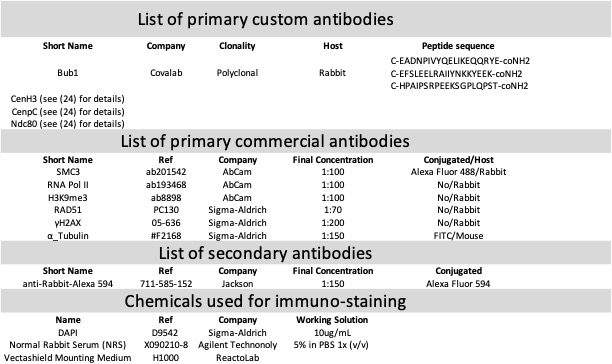


**Table B**: Antibodies and chemicals used. A combination of custom and commercially

available antibodies were employed for the immunostaining assays.

**
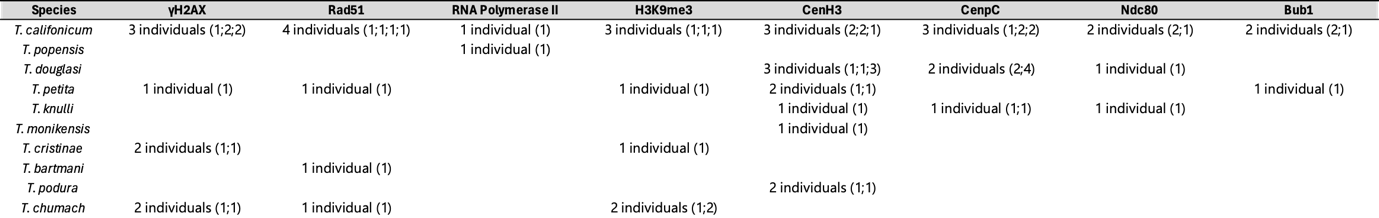
 Table C:** Inter- and intra-species replication of the cellular patterns described in this study as well as in (24, 25). The number of technical replicates for each individual are indicated between brackets and separated by semi-columns.
