# Supplementary material for: Dynamics of recombination, X inactivation and centromere proteins during stick insect spermatogenesis
Source: PLoS Genet. 2025 Aug 18;21(8):e1011827. doi: 10.1371/journal.pgen.1011827 (PMC12393702; doi:10.1371/journal.pgen.1011827)
Supplement: Attachment — Submitted filename: Re-review_Dumas et al_PLOS Genetics_2025.pdf [file pgen.1011827.s004.pdf]

The authors have addressed most of my concerns, and the new version of the text is more streamlined and easier to read compared to the original version. I feel that once the following minor comments are addressed, the manuscript is suitable for publication in PLOS Genetics.

Minor comments:

- Authors need to be consistent with recombination wording when referring to Rad51. While the authors are correct in their rebuttal that Rad51 is often used as a marker of recombination, it marks recombination INITIATION, and stating "recombination happens before synapsis" makes it sound like recombination is COMPLETE before synapsis, which is impossible. The authors should consistently say "recombination initiation", when referring to Rad51 foci.
    - Ex - Line 122 of p32, "homologous recombination" → "INITIATION of homologous recombination"
    - Ex - Line 364 of p42: Change "recombination" → "recombination INITIATION"
- Antibody staining in figures is still too weak and hard to see, especially in the merged images. All channels need to be made brighter, and it would be very helpful for the reader to show 3-way merges between all channels in all figures.
- Line 72-73 of p30: Change "In most eukaryotes," to "In many eukaryotes," Holocentricism is probably under-appreciated in plants and insects, which greatly outnumber vertebrates, due to the low number of species actually studied.
- Line 86 p30, "they also pose significant challenges for preserving genome integrity": Please describe how and what kind of challenges.
- Incorrect referenced figures:
    - Line 146 of p33, "SMC3 filaments become interrupted near chromosomal ends": This is only clearly shown in Supplementary Figure 3A'-O' with the inserts. Other figures should be not referenced here, or it needs the same inserts as in Supp Fig 3.
    - Line 308-309 of p39-40: Leptotene and zygotene stages are referred here. Only Figure 6A-J, Figure 7A-E, Figure 8A-E should be referenced here.
- Line 152-153 of p33, "two *Timena* species forming one "ring" bivalent": Do these two species ALWAYS have one ring bivalent? How often was the ring bivalents observed?
- Line 163 of p33, "evidence": Antibody validation concerns. While I am not concerned about the SMC3 antibody (chromosome axis in meiosis has an unmistakable structure), the foci of H2AX and Rad51 are less convincing. The Rad51 seems more real than the H2AX signals, as the signal seems to be a bit more specific, but the authors really need to include a western blot or some other kind of validation for these antibodies, such as Bleomycin test that will show more of both H2AX and Rad51 after bleomycin treatment if that's real signal. I do not see any validation of the antibodies, even in the other papers mentioned.
- Line 182 of p34, "compared to both insects and mammals": *Timena* is an insect too. Change to "compared to other insects and mammals"

- Despite the characterization of spermatogenic stages in the methods, it is still not clear to me how leptotene and zygotene are distinguished here… for example - Line 206-207 of p35, "(i.e., leptotene and zygotene; Figure 3A-j)": To me, these look basically the same.
- Line 211 of p35, "(Figure 3a-e', etc.)": Confusion about the two cells in a vs a'. Why are two different cells shown and why does only one cell have an "X body"?
- Some text in the results section need to be moved to the discussion:
    - Line 227-262 of p36-38
    - Line 333-335 of p41
    - Line 340-350 of p41, "Initially, CenH3…."
    - Line 351-356 of p41
- Line 290 of p39: Change "all *Timena* species" → "all EXAMNIED *Timena* species"
- Line 366-368 of p42, "… but absent in only *D. melanogaster* and *C. elegans*. It may also reflect the ancestral state in insects. Indeed, only in female meiosis of *Drosophila* (Diptera order) is synapsis known to precede recombination.": These sentences are unclear and seemingly redundant.
- Line 370-372 of p42, "*Bombyx mori*": There still seems to be a lack of understanding regarding recombination initiation and completion. Recombination nodules observed in *B. mori* by EM after synapsis indicate the recombination doesn't COMPLETE until after synapsis. There is currently no data on recombination initiation and pairing or synapsis in Bombyx or other moths.
- Line 428-429 of p43-44: Senaratne *et al.* (2021) should be referenced here.
- Supp Fig 4: Arrows marking X chromosome should be included in all panels, not just DAPI panel
- Supp Fig 4, line 687 of p61: Missing/mislabel "H, I, J"
- Supp Fig 4, line 687 of p61: Missing description of "SMC3" in figure legend
- Typos:
    - Line 159 of p33: Typo in "recombination-dependent machinery" → "recombination machinery-dependent"
    - Line 183 of p34: Typo in "as" → "at"
    - Line 441 of p45: Typo in "Cenh3" → "CenH3"
    - Line 635 of p55: Typo in "(a-o)" → "(a-j)"
