# Supplementary material for: Dynamics of recombination, X inactivation and centromere proteins during stick insect spermatogenesis
Source: PLoS Genet. 2025 Aug 18;21(8):e1011827. doi: 10.1371/journal.pgen.1011827 (PMC12393702; doi:10.1371/journal.pgen.1011827)
Supplement: Attachment — Submitted filename: Response to Reviews.doc [file pgen.1011827.s003.doc]

*General author’s response:* *Given that our results describe three distinct processes occurring during spermatogenesis, we initially believed that integrating a short introduction before, and a discussion immediately after, presenting the observations would enhance clarity. However, we now recognize that this structure is at the root of the reviewers' main concerns. In the revised manuscript, we have restructured and rewritten sections following the conventional article format, with clearly separated introduction, results, and discussion. Moreover, aspects considered to be speculations are now clearly reported results and distinguished from interpretations.*

Point-by point response to reviews

**Reviewer: 1**

**Reviewer #1: ASSESSMENT**

In this manuscript, Dumas & Toubiana et al. beautifully illustrate the dynamics of spermatogenesis in Timema californicum. With clear, interpretable cytology, the authors describe the timing of homologous recombination in meiotic prophase, the dynamics of X chromosome inactivation, and the association of centromere proteins with chromosomes. Their results suggest that the initiation of recombination precedes cohesin filament thickening and that RNA polymerase II is excluded from X chromosomes throughout spermatogenesis (despite extensive occupancy on autosomes). They also investigate the distribution of other centromere and kinetochore proteins to determine whether they also follow (spoiler, they do not) the unusual CenH3 deposition pattern that these authors described in a recent preprint. These results represent some of the first molecular cell biological investigations into spermatogenesis in Timema, contributing to our understanding of how meiosis has evolved.

Although this is an exciting study of a non-traditional model organism, the manuscript has some issues. Because the manuscript does not have line numbers, I have included a snippet of text or indicated the general region of the manuscript I’m describing for each comment.

*Author response: we thank the reviewer for their constructive evaluation of our work. The manuscript was revised thoroughly to address the points raised (see point-by-point replies below). We now also added line numbers to facilitate re-review.*

MAJOR COMMENTS


The results are partially redundant with those of an unpublished study.
- The results on longitudinal CenH3 loading recapitulate recently preprinted findings (Toubiana et al., 2024; <https://doi.org/10.1101/2024.06.30.601385>), but the authors do not clarify what are the unique results of this study with regard to CenH3. Such clarification would be helpful.

*Author response: For clarification, we now emphasize the new results based on additional centromere proteins and spermatogenesis stages examined and notably the question of how conserved the previously reported, unusual CenH3 phenotype is among Timema species. Specifically, we describe in lines 279-282 “… we extended our previous observations on CenH3 to include other centromere proteins and additional stages of spermatogenesis (i.e., not examined in (25))”. In our previous findings, we describe the CenH3 loading during meiosis 1 and the distribution of 2 kinetochore proteins at metaphase 1. The present study extends the description of CenH3 distribution to meiosis 2 and spermiogenesis, while describing the distribution of the 2 kinetochore proteins, and a protein from the spindle assembly checkpoint, throughout spermatogenesis. We also precise in lines 286-288 that our previous observations focused on a single species (i.e., T. douglasi), while our new study extends across the Timema phylogeny. Finally, we provide new findings on the relationship between transcription and centromere protein distributions throughout spermatogenesis and refer to these in the main text, as well as in figures and supplementary figures, at the result section (lines 272-310).*

,
Antibody Validation
- The authors do not describe any steps taken to validate the use of these antibodies in these species. To ensure that the antibody signal is specific to the protein of interest, best practices include validating antibodies via the absence of staining in knockout animals or some similar experiment. I understand the challenge of performing such experiments in a non-traditional model, and I am not asking for such near-impossible experiments to be performed. However, it could be nice for the authors to describe their rationale for trusting each antibody in the Methods section.

*Author response: we now added a section in Material and methods (“Design, selection and validation of antibodies”) that explains how the antibodies were selected and the reasoning for their validation in our study. This validation considers the cytological patterns observed in other organisms, the previous validations in Timema species (notably published in the study cited above), as well as the specificity and immunoreactivity of the custom antibodies via ELISA assays.*

Replication
- The number of replicate examples of each phenomenon is not reported, and only single images per figure (and per species) are given. It is difficult to assess the generality of the authors’ conclusions without this information.

*Author response: we now added in Supplementary table 3 the number of individuals and technical replicates (i.e., number of staining per individual) for each species and antibody. Furthermore, we discuss the limitations of the study (especially regarding the consistency of the results across species and stages) in the section “Limitations of the study” in Material and Methods.*

Viability of reagents across species
- The authors should clarify whether some of the variability they see across species could be due to the variable effectiveness of their antibodies. For example, the inconsistent detection of yH2AX across species during pachytene, variable retention of H3K9me3 through spermiogenesis, could ostensibly be due to variable binding efficiency of this antibody.

*Author response: We acknowledge in lines 188-190 and 222-225 that we cannot formally exclude that the variability across species is caused by variable antibody affinities. Yet, we also precise that some cytological patterns, such as the cloud of γH2AX foci at a polarized region of the nucleus during zygotene or the H3K9me3 coating of the X chromosome during pachytene, are observed in all Timema species. These observations suggest that the variability observed across species is not caused by variable antibody affinities.*

Clarity of writing
- The introduction is lacking specifics—I suggest detailing what is known from other systems about the features of meiosis that are studied here. This will prepare the reader for understanding your results.
- It should be clarified what is meant by “XO system” in parentheses.
- It should be made clear what methods were used to obtain the results from the “Toubiana et al., 2024” and “Jeli’s Paper”. These results should also be described in more detail.
- One interpretation is confusing and needs further explanation. “Importantly, this step seems to require the presence of Rad51…and does not seem to necessitate the accumulation of yH2AX on the X chromosome.” The authors should explain the evidence for these claims.

*Author response: Following these comments, we extended the introduction and detailed the diversity observed in other organisms for all the cellular processes that we analyze in this study.*

*We also clarified that XO system means the absence of Y chromosome (line 58) and the interpretation about the necessity of Rad51 but not γH2AX for X chromosome inactivation (lines 181-188 and 233-240). We also apologize for erroneously uploading an incompletely formatted version of our manuscript for review. By the time we revised the manuscript, the results from “Toubiana et al., 2024” and “Jeli’s Paper” were published and we now refer to them as actual citations (citations 24 and 25 in our revised manuscript).*

Insufficient background information is given
- Much of the novelty of the paper lies in describing the ways in which Timema spermatogenesis differs from “canonical meiosis”. But for readers who aren’t intimately familiar with what occurs in “canonical meiosis”, it will be hard to know what is different in Timema. I list below several locations in which the authors should highlight what is known and how it differs.

*Author response: Indeed, and as explained above, we extended the introduction and the discussion to specifically address what is canonical and what is not among the meiotic processes that we investigate in this study. See also the precisions to each concern below.*

o Abstract: “Our results reveal that in males, …. bind locally or longitudinally along the chromosomes.” The authors should clarify: 1) does the initiation of recombination before synapsis differ from canonical meiosis? Or is this consistent with canonical meiosis? 2) Is X chromosome silencing different from or consistent with canonical meiosis?

*Author response: the conservation of the relationships between recombination and synapsis was clarified in lines 364-371 and we added “(unlike Drosophila meiosis)” in the abstract. For the X chromosome inactivation, we clarified the conservation of the Timema patterns in lines 174-181, 236-240, 391-393, 404-406 and 412-414.*

o Please clarify if it is “novel” or “canonical” that the yH2AX and Rad51 cloud of foci is found in a polarized region of the nucleus.

*Author response: This pattern is observed in grasshoppers and a true bug, which suggests that this pattern is conserved across the polyneoptera and condylognatha insect orders. This is clarified in lines 133-136.*

- In the Results and Discussion, the authors introduce several experimental approaches and concepts without giving sufficient background for non-expert readers to understand.
o SMC3 filament thickening—this has not been introduced.

*Author response: SMC3 marks chromosomal axes but can also be used as marker for the progression of synapsis as SMC3 filaments become thicker as chromosomes pair and synapse. This is now explained in lines 124-126.*

o What yH2AX and Rad51 label and why this is important for analysis of crossover recombination.

*Author response: γH2AX labels the formation of programmed DNA double-stranded breaks (DSBs) that rapidly induce the phosphorylation of the histone variant H2AX (γH2AX). The recombinase Rad51 is involved in homologous recombination and mediates the single-strand invasion to repair DNA after DSBs. This is now detailed in lines 118-121.*

o Cross-shaped SMC3 labeling indicating single terminal or sub-terminal chiasmata and ring-shaped bivalents indicating two chiasmata.

*Author response: Interruptions of SMC3 labeling during metaphase I often highlight chiasma sites that correspond to the contact zone where homologous chromosomes remain connected. The relationship and reasoning between SMC3 labeling and the number of chiasma sites is now detailed in lines 145-155.*

o Briefly clarify the role of RNA pol II before discussing its staining pattern.

*Author response: RNA polymerase II phosphorylated on serine 2 is a conserved transcriptional marker of transcription elongation. This is clarified in lines 201-203.*

o Clarify what the correlation is: “to probe for a broader correlation between transcription and centromere protein recruitments as seen in C. elegans (36) and some Lepidoptera (37),”

*Author response: this section has changed and is now formulated “It is only during the transition from zygotene to pachytene that cells establish the expected monocentric distribution of CenH3 on the autosomes but not on the X, which we here show occurs in parallel to the activation of transcription from the autosomes and silencing of the X (Figure 3K-O; Figure 5K-O; Supplementary figure 5). Considering this parallel occurrence, we examined whether a broader correlation could exist between transcription and the recruitment of centromere proteins in Timema.”*

o It is odd that the authors’ recent transcriptomic study (Djordjevic et al; <https://doi.org/10.1101/2024.07.01.601468>) is not referenced in the MSCI section. Those results are pertinent to this study.

*Author response: this is now referenced in lines 198-199.*

o It is not clear until late in the manuscript how the authors identify the sex chromosomes in their cytology. This should be clarified the first time analysis of sex chromosomes is mentioned.

*Author response: this is now clarified in lines 169-172 (i.e., the first analysis of the X chromosome). Specifically, the X chromosome was previously characterized by cytological and ChIP-seq data on CenH3 and H3K9me3 (1, 2). This allowed us to further characterize the X chromosome as the chromosome with intense DAPI staining in the nucleus.*

Organization of figures—Figure 5 is a cartoon summary of the results that are presented in Figures 3 and 6 and in a “previous study” (Toubiana et al., 2024). It would make more sense to me to swap its position with Figure 6.

*Author response: because Figure 5 is a cartoon summary (“graphical abstract”) and not an actual result per se, we use it as Supplementary figure 5 in the revised manuscript.*

SIMPLE FIXES

Citation Issues
- Several citations appear to be missing. In the introduction, the authors cite “Toubiana and Jeli’s papers”, and later they cite “Jeli’s Paper” and “Toubiana et al., 2024” separately, although these are not included in the manuscript list.

*Author response: this is fixed and all citations are properly referenced.*

- A reference is required for this claim: “These meiotic variants linked to recombination are notably observed in insects…”

*Author response: this statement has been removed from the revised manuscript.*

- A reference is required for this claim: “Dosage compensation in somatic tissues equalizes transcription levels between the single X copy in males and the two X copies in females”

*Author response: this statement has been removed from the revised manuscript.*

- In the introduction, the authors write that “meiosis and spermiogenesis are conventionally viewed as highly conserved processes, partly because of conservation in model organisms”. I think people already appreciate the many differences between traditional model organisms in meiotic processes and understand that inference can be drawn from both conserved and variable aspects of meiosis and spermiogenesis. Two reviews come to mind just for pairing (<https://doi.org/10.1038/nrg1614>) and meiotic recombination machinery (<https://doi.org/10.1038/s41576-023-00669-8>)
*Author response: we rephrased this statement by “While traditionally viewed as conserved across model organisms, emerging studies reveal significant variation in meiotic and post-meiotic processes underlying sperm maturation” and added the suggested citations in lines 40-42.*

Wording
- “The transition from diploidy to haploidy is conditioned by meiosis, a cellular process…”—consider changing “conditioned by” to “occurs via” or some other substitute.

*Author response: we changed the wording as the reviewer suggested.*

- Change “maturate” to “mature” in the sentence introducing spermiogenesis.

*Author response: we changed the wording as the reviewer suggested.*

- Change “sperm formation” to “spermatogenesis”

*Author response: we changed the wording by “sperm maturation”.*

- Change “as it is the case” to “as is the case” when describing achiasmate male meiosis in Drosophila.

*Author response: we changed the wording as the reviewer suggested.*

- Change “These meiotic variants linked to recombination are notably observed in insects” to something like “Although much variation in the timing of recombination has been documented in insects”

*Author response: we changed the sentence by “In organisms that do recombine, the temporal relationships between meiotic recombination and synapsis can also vary, with dependence or independence of synapsis on the occurrence of double-stranded breaks (DSBs) and recombination.”*

- CenpC is written as CenPC in Figure 7, but written as CenpC elsewhere.

*Author response: we corrected this.*

**Reviewer #2: Summary:**
This manuscript was focused on the meiosis in the relatively unexplored stick insect group Timema. While the main species studied was T. californicum, other species in this group were explored as well. Using immunofluorescence, key meiosis discoveries were explored. The first major discovery was the idea that recombination precedes synapsis in Timema, which was shown by the presence of yHAX and Rad51 before the thickening of the cohesion protein SMC3. The second main discovery was that the X chromosome remained silent in all Timema species, despite two rounds of transcription activation. This was shown through the coating of H3K9me3 on the X chromosomes during both rounds of transcription upregulation using the presence RNA Pol II as a reference for increased transcription. Lastly, this paper showed that while a single centromere protein CenH3 was distributed longitudinally before the first meiotic division, it became monocentric afterwards. Kinetochore proteins CenpC is added in a monocentric fashion, and then shortly before the first meiotic division Ndc80 and bub1 – two outer kinetochore proteins – are also added and shown to be monocentric. These three discoveries do a good job highlighting key aspects of meiosis in a non-model organism.

Summary of overall impression:
This paper does a fantastic job introducing key meiosis discoveries in an understudied and non-model group Timema. It utilizes beautiful images with solid photo proof of its claims. The experimental design is solid from the standpoint of a non-model organism – extremely clear and provides proof of the meiosis claims the authors stated. I especially liked figure 5, which did a good job explaining the second half of the paper. Overall, I didn’t have suggestions for new experiments, but there needs to be fixes in references, figures, and how you discuss your findings. These problems may involve an extra check of the submitted manuscript, but do not detract from the findings of the paper.

*Author response: We thank the reviewer for their positive evaluation of our work. We have integrated all the suggestions and answered the few concerns of the reviewer (see points below).*

Notes for review:
Overall observations:
- References are not numbered properly, and they are missing references.
The manuscript references to papers I cannot locate online: Toubiana et al., 2024.

*Author response: this was unfortunately caused by a wrong version sent to the journal and we apologize for the frustration caused. We realized this mistake and contacted the journal, but it was already not possible to exchange the manuscript versions. We now have implemented the missing references and properly referenced the citations that could not be found online (e.g., Toubiana et al. 2024).*

- The species involved in this research have an XO system. While fascinating, the paper makes many references to a mice model (XY), and the authors need to stress more that it might not be appropriate to compare their system to mice which may not match their system correctly.

*Author response: In the revised manuscript we extended our comparisons to other model systems, such as grasshoppers, hemipteran or chicken, which have different sex chromosome systems including XO systems. We also clarify the key aspects of the different sex chromosome systems where appropriate (e.g., lines 179-181).*

- Is there any other paper which ncd80 isn’t loaded late? I would like some paper references showing that this is a normal occurrence in most species.

*Author response: unfortunately, the literature regarding the timing of deposition of the Ndc80 protein during meiosis is limited to yeast, mouse and C. elegans. We precise the limited number of species in which it was studied in lines 318-321, and add the associated references.*

- Some in-text citations are not properly formatted.

*Author response: this was due to the error in the version sent to the journal, as explained above. This is now fixed in the revised manuscript.*

Specific changes:
- “Indeed, only in female meiosis of Drosophila (Diptera order) is synapsis known to precede recombination”
o C. elegans also have this system

*Author response: This correction was added in lines 364-366.*

- One claim says that CenH3 localization pattern is over the entire length of the chromosome in all Timema species, i.e longitudinal distribution, in early prophase I. Then in later prophase I it switches to a monocentric distribution. This is shown to be true, and it is argued that there is a significance of the switch from longitudinal to monocentric distribution. However, based off of Figure 6 images, it appears as though the CenH3 longitudinal pattern is less bright than the foci seen in the images in the mid-zygotene panel. This implies to me that the cell may know where the real centromere is located even in early stages, and this pattern holds less significance than previously stated.

*Author response: Indeed, we can observe in mid-zygotene that CenH3 signal is very bright at specific foci on each chromosome, which are presumably the regions where centromeres are defined and the other centromere proteins assembled. This is detailed in our recent paper (2) where we show that Timema chromosomes are characterized by functional monocentromeres despite the longitudinal deposition of the CenH3 proteins. We do not emphasize the mentioned CenH3 signal to avoid giving the false impression of this being a new discovery. In the present study, we characterized the dynamics of CenH3 deposition throughout spermatogenesis and how it correlates with transcription and the spatiotemporal deposition of other centromere proteins. We thus refer to the switch being “significant” (although we do not use this exact term) in the autosomes with regard to the activation of transcription, not with regard to centromere identity.*

- Supplemental figure 3:
o I could not accurately see any chiasmata in this figure on the rods but was able to see one in the ‘ring’ on the terminals. Perhaps there is a better way to demonstrate chiasmata in the rod formations?
o I would like to know the number of chromosomes in all Timera species mentioned in paper, so I know better what I’m looking at the metaphase I stage.

*Author response: We rephrased this section and explained that SMC3 filaments become interrupted near chromosomal ends during metaphase I, which may highlight chiasma sites (lines 145-148). We also zoomed onto some chromosomes to better show the regions where SMC3 filaments are interrupted. Furthermore, we added to the figure the number of chromosomes for each Timema species (determined in previous karyotype studies).*

- Figure 4 / Supplementary Figure 4:
o I would also like to know how much heterochromatin is on the X chromosome to confirm that there is indeed X inactivation occurring and not just an abundance of heterochromatin.

*Author response: we quantified the amount of heterochromatin on the X chromosome in a recent paper via H3K9me3-directed ChIP-seq experiments and by showing (in RNAseq datasets) that the X chromosome has strongly reduced expression in male gonad (1). We now refer to this study in our revised manuscript (lines 249-251).*

- Supplemental figure 4:
o In these images (T petita and T cristinae) there are bright foci that are different from that which coat the X. These are presumed to be heterochromatin, but they appear to be rather large. I would like an explanation regarding the size and distribution of heterochromatin to better understand these images. I believe that Pol II provides better evidence of X inactivation than H3K29me3.
o Furthermore, everything is a little bit small in this image, what am I looking at?

*Author response: Indeed, we observe large patches of H3K9me3 signal elsewhere than on the X chromosome. However, no clear explanation(s) can be given for now. These patches may highlight chromocenters as in Drosophila (3) or some other chromosomal regions that are highly heterochromatic. In the revised manuscript, we suggest that these large H3K9me3 patches may indicate clusters of heterochromatin regions (lines 256-259). We also zoomed on the images and added the cellular stages for the different nuclei observed.*

- “This is revealed by the fact that the Timema X chromosomes maintains its condensation state and transcriptional inactivation long after the first meiotic division […]”
o I would like to not just see like of RNA Pol II at these later stages but also show H3K9me3 as well

*Author response: we added the spermiogenesis stages for T. californicum in the main figure 4 and precised the meiotic and spermiogenesis stages associated with the H3K9me3 signal for the other Timema in the Supplementary figure 4.*

- Supplemental figure 5: The images label shows Rad51, but the figure caption states CenH3

*Author response: this is now corrected.*

- Supplemental figure 6: DAPI and CenH3 labels need to be switched.

*Author response: this is now corrected.*

- Figure 7: Fix CenpT capitalization.

*Author response: this is now fixed.*

- Supplemental Figure 7: There are no images above the figure caption.

*Author response: this is now corrected.*

**Reviewer #3:** The manuscript entitled, “Dynamics of recombination, X inactivation and centromere proteins during stick insect spermatogenesis” by Dumas et al. utilizes cytological approach to investigate the cellular patterns and dynamics of chromosomes during meiosis in Timema californium and other Timema species. This study describes results in three separate areas: recombination initiation before synapsis, meiotic sex chromosome inactivation (MSCI), and centromere proteins during chromosome segregation.

Overall, the manuscript presents interesting results that would be of interest to the readers of PLOS Genetics. However, the major findings in this paper feel somewhat disconnected, and many of the “results” are, in fact, speculation. The paper also lacks any true mechanistic insights. Still, it is important to explore biology in new model systems and I do think this paper could have a home in PLOS Genetics. However, the paper requires major revisions before being considered for publication in this journal.

*Author response: we thank the reviewer for their constructive comments and greatly appreciate the time and effort invested to review and improve our work.*

*As a general comment, we acknowledge that our choice to format the manuscript with combined results and discussion sections, along with separate introductions for each topic (i.e., recombination timing, X chromosome inactivation, and centromere protein distribution), has led to confusion. This structure indeed blurred the distinction between our findings and subsequent interpretations. We have rewritten our manuscript with clearly separated introduction, results, and discussion sections to ensure greater clarity and address the reviewers’ concerns effectively.*

*Furthermore, we agree that our paper is about investigating cellular patterns and not detailed molecular mechanisms. We thus changed or removed all statements related to specific mechanisms in the revised manuscript.*

Specific concerns:

1. The manuscript would benefit from an expanded “Introduction” section to help tie the results together more, and the addition of a separate “Discussion” section to more clearly separate speculation from actual findings. In the introduction, the authors should elaborate on what is known about recombination, MSCI, and centromere proteins during meiosis in Timema and in other species. The authors cite several papers throughout the manuscript, especially Toubiana et al. (2024) and another biorxiv paper (which is often informally referenced only) from this lab, the results of which need to be described in the intro.

*Author response: the rewritten manuscript includes now an expanded introduction that better explains what is known and the diversity encountered for each of the three processes described in this study. Furthermore, we added a separate “Discussion” section and properly referenced the two biorxiv papers from our lab, which have now been published in Science Advances and PLOS Genetics.*

2. As mentioned above, there is lots of speculation throughout the results section. The authors either need to dramatically rephrase the text or do additional experiments to support their conclusions. Some specific examples:
- Last sentence of second paragraph of results section – “… the condensation of chromosomes via loop extrusion..” … What is the evidence for this? The authors have not, even a little bit, showed evidence of loop extrusion. I would remove this statement.

*Author response: this statement was referenced with published citations, not our own observations. We regret any misunderstanding and acknowledge that a separate discussion section would have prevented this confusion. Regarding the reference on loop extrusion for chromosome condensation, we decided to remove this statement as not critical for the general understanding of the manuscript.*

- First paragraph after Supplemental Figure 1, “Finally, we observed one to two crossovers per chromosome as indicated by the chromosome shapes as well as shape of SMC3 filaments at metaphase I.” This is speculation. Clearly the authors observe rod or ring-shaped bivalents, which MAY be the result of COs, but things besides COs can hold homologs together at metaphase I (as in flies and C. elegans, for example). The authors either need to look directly at crossovers using cytological or sequencing-based methods, or remove any mention of “observed crossovers” from the paper, since there is no actual data on this.

*Author response: we have changed the phrasing of this section to state “… the terminal positions of chiasma sites suggest that crossing-overs may preferentially occur on chromosomal ends in Timema males, although such conclusion requires further molecular evidence.” We would also like to emphasize that the number of COs per chromosome is not central to our main conclusions on recombination timing, which is the focus of this section.*

- Paragraph before Supplemental Figure 3 – the authors say that in Timema, recombination precedes synapsis. Again - speculation. The authors have shown that DSBs form before synapsis, but they have not shown any evidence of recombination or when it occurs. Again, the authors either need to look directly at recombination or crossovers, or reword this section.

*Author response: our conclusions are not solely based on DSBs formation (evidenced by γH2AX signal), but also on the signal of the recombinase Rad51, which occurs before synapsis (Figure 2; Supplemental figure 2). Rad51 mediates the single-strand invasion to repair DNA after DSBs and is a well-known marker for the initiation of recombination in mammals and insects. Furthermore, DSB and early recombination markers are widely used across eukaryotes to assess the timing of recombination in relation to synapsis (see (4, 5) among others). We explained more clearly the roles associated with γH2AX and Rad51 signals, and our reasoning to interpret the timing of recombination in regards to synapsis (lines 118-126).*

- Paragraph after supplemental Fig 3, first sentence (“We then Investigated…”) – this is written as if these findings are part of this study, but as far as I can tell, they are not. Is this describing findings from a previous study? There is no citation. How did the authors come to this conclusion? Please cite if referencing results from a previous study or remove if implying these conclusions were drawn from the work presented in this manuscript.

*Author response: our study expands upon previous work, which identified that a reduced X expression in males at the 4th nymphal stage is associated with an absence of polymerase II signal in pachytene. The current study examined the dynamics of transcription and sex chromosome inactivation throughout spermatogenesis, not just in pachytene. Figure 3 and Figure 4 newly document Polymerase II and some MSCI markers (i.e., H3K9me3 and sex body) dynamics from leptotene to mature sperm. Overall, this analysis allowed us to uncover that the X chromosome remains condensed and transcriptionally silenced throughout spermatogenesis, despite two rounds of genome-wide transcription; a finding that was not known before.* *These points are now clarified given the overall new structure of the manuscript and clear distinction of previous and new results versus interpretations.*

- First paragraph after Supplemental Figure 4, “Finally, the fifth and last step involved the loss of MSCI and transcriptional markers in all species as spermatids elongate and maturate.” It is not clear how the authors came to this conclusion, and thus it feels like speculation. Please explain the data supporting this conclusion or remove.

*Author response: this sentence is not speculative and refers to observed loss of MSCI (H3K9me3 and sex body) and transcriptional (Polymerase II) markers from the elongated spermatid stage (Figure 3, Figure 4 and Supplementary figure 4). We have revised the phrasing, added references, and specified these stages in Supplementary figure 4 for clarity (lines 259-262).*

- Same paragraph after Supplemental Figure 4, the authors need to cite the findings supporting each proposed step described in this paragraph if they are from the present study or from other studies previously published (there is not data in this manuscript to support these conclusions). Please cite if referencing results from a previous study or remove if implying these conclusions were drawn from the work presented in this manuscript.

*Author response: All the proposed steps are described based on the present study. We give figure references as support for each of these steps. Furthermore, when referring to previously published works, we clearly state so and indicate the relevant citations (see lines 227-262).*

- Related to all the above – the authors should create a discussion section (currently missing from the manuscript) and clearly state these speculations as just that.

*Author response: a discussion section has been added to the manuscript which indeed clarifies the distinction between our new findings and more speculative suggestions. Specifically, the discussion now serves to 1) address the future directions regarding the evolution of the timing of recombination, 2) speculate on the different hypotheses that may explain the dynamics of MSCI on the X chromosome of Timema, 3) speculate on the mechanisms involved in the deposition and removal of CenH3 in Timema.*

3. When looking at H2AX staining (and throughout the manuscript), how do the authors know which chromosome is the X? A more detailed explanation of meiotic staging and chromosome identification is required, at least in the methods if not in the main text. Ideally, I would like to see DNA FISH of some to confirm identification of the X chromosome throughout meiosis.

*Author response: We added a specific section “Characterization of spermatogenetic stages and X chromosome identification” in the material and methods to explain how we can discriminate between different meiotic and spermiogenesis stages, as well as how we can identify the X chromosome. Furthermore, we refer to this section in lines 128-129 and 171-172, when we respectively start referring to meiotic chromosomes and the behavior of the X chromosome in Timema.*

4. Supplemental figure 4 (as an example - but many other supplemental figures) – these large fields of cells need more direction for the reader. What stages are we looking at? Zoomed insets clearly showing what the authors want to emphasize are needed throughout.

*Author response: As requested, we limited these large fields of cells to a more restricted and fewer number of cells for clarity, added cellular stages to all supplementary figures and zoomed insets when needed.*

5. It would be very helpful and would make all figures more convincing if the authors would take DAPI-stained images of whole-mount testes, clearly labeling the different stages of spermatogenesis so that readers can better understand the cells in their native context. For example, the images of elongated spermatid nuclei – out of the context of the gonad, this looks just like any other little nucleus to me. How do the authors know this is an elongating spermatid? Tubulin staining would also help.

*Author response: The supplementary figure 3 was made to label the different stages of spermatogenesis based on DAPI, SMC3 and tubulin stainings to illustrate how staging is done in Timema. Timema testes exhibit a grape-like appearance, with the shoot containing mature sperm cells and the grape forming an oval and opaque structure encompassing cells in various meiotic and spermiogenesis stages. This prevents a clear labeling of the different stages from whole-mount testes. We thus performed our stainings in squashed tissues, where cells are individualized as a monolayer. A picture of this organ can be found in (2).*

6. First paragraph after Supplemental Figure 4, “During the first step, the polymerase II and MSCI markers are absent from nuclei in leptotene and zygotene stages, …” Regarding “MSCI markers are absent in zygotene”, there seem to be some signals in Figure 4B. It is unclear how the authors made their conclusion that MSCI markers are absent. Please explain or correct.

*Author response: H3K9me3 labels heterochromatin and is therefore also a marker of MSCI (as shown notably in mammals (6) and stick insects (1). However, not all H3K9me3 signals are indicative of MSCI. For example, autosomal regions such as peri-centromeres are generally known to be heterochromatic and thus marked by H3K9me3 without experiencing MSCI, although this is not the focus of this study. The patches of H3K9me3 in Figure 4B are presumably highlighting some of these autosomal regions as the X chromosome is not yet condensed or marked by other markers of MSCI initiation such as Rad51. To avoid any misunderstanding, we rephrased this sentence in the text as “During the first step, the polymerase II is absent from nuclei in leptotene and zygotene stages, and all chromosomes, including the X chromosome, are most likely transcriptionally inactive (Figures 3A-J).”*

7. Last paragraph on p24, starting “The mechanisms engaged in MSCI in Timema offer a new perspective..” – the authors have not shown ANY mechanism in this paper. Please remove this statement.

*Author response: We replaced this sentence by “… the persistence of a complete transcriptional inactivity of the X chromosome throughout Timema spermatogenesis questions the driving forces behind the origin of the MSCI process.”*

8. As the study used custom antibodies and antibodies generated for mammals, the authors need to include some type of antibody validation (such as Western blot showing a band of the correct size), especially for Bub 1, but also for H2AX and Rad51.

*Author response: This comment was also raised by the first reviewer and we now have added a section in Material and methods (“Design, selection and validation of antibodies”) that explains the reasoning for the validation of our antibodies. For example, we have indeed validated our custom CenH3 via Western blot in our previous study (2). For antibodies where this was not done, we further consider the specificity and immunoreactivity of the custom antibodies via ELISA assays, the conservation of the cytological patterns in other organisms, and the consistency of the pattern within and between species.*

Minor suggestions:

1. Supplemental figure 6: Missing legend for the bottom images for T. bartmani. Please describe as it is unclear what the authors trying to show there (row 3).

*Author response: this was to show the early prophase I stages with CenH3 distributed longitudinally on all the chromosomes. For clarity, we kept T. podura only in the revised manuscript as it is from the same clade as T. bartmani and both species display the same CenH3 patterns during spermatogenesis.* *We also clarified the corresponding legend.*

2. Missing Supplemental Figure 7

*Author response: this is fixed.*

3. The signal in all images (especially the magenta signal) needs to be brighter to be seen. Difficult to see even on a computer. The authors should adjust brightness/contrast, at least in a supplemental figure. Also zoomed insets would be helpful for almost all figures instead of arrow heads.

*Author response: we adjusted brightness and contrast to improve the brightness of the signal and added zoomed insets.*

4. Figure 2 legend: Typo “…… meiocytes (A-Zd) and spermatids (a-j), at……”

*Author response: this is fixed.*

5. Supplemental figure 3B: Legend indicated “5 Timema species”, but only three species were shown in the figure

*Author response: this sentence was replaced by “(A’-L’) illustrate rod and ring bivalents across 4 Timema species at metaphase I.” As indicated in the figure, the 4 species are T. californicum, T. douglasi, T. podura and T. bartmani.*

6. Supp Fig 4 – what are foci in S4 top row? They seem like specific signal to me…

*Author response: This comment was also raised by the second reviewer, and no clear explanation(s) can be given for now. In the revised manuscript, we suggest that these large H3K9me3 patches may indicate clusters of heterochromatin regions (lines 255-258) but we currently have no data that allow us to characterize them. These patches may highlight chromocenters as in Drosophila (3) or some other autosomal regions that are highly heterochromatic. Further H3K9me3-directed ChIP-seq experiments may help characterizing these autosomal regions in the future.*

7. Supplemental figure 5: Mislabeling “CenH3” in top panel

*Author response: this is fixed.*

8. Supplemental figure 6: Mislabeling “CenH3” and “DAPI” in top panel

*Author response: this is fixed.*

9. Figure 7: Typo “CenpC” in top panel

*Author response: this is fixed.*

10. Figure 7 legend: Typo “…… meiocytes (A-Zi) and spermatids (a-j), at……”

*Author response: this is fixed.*

11. Include CenH3 and kinetochore antibodies (CenpC and Ndc80) in Supplemental Table 1 and cite the source.

*Author response: this is done.*

12. Regarding the centromere localization in meiosis here – starting off as chromosome-wide and then becoming more monocentric as meiosis progresses - A recent PLOS Gen publication in moths (Hockens et al) showed something similar for CENP-T, and this should be referenced.

*Author response: this is now referenced in lines 449-452.*

**Additional Editor Comments (if provided):**

**additional reviewer comments:**

Review for Dumas et al., 2024 – *PLOS Genetics*

In this manuscript, the Dumas and colleagues provide insight into the conservation of spermatogenesis events in the stick insect group *Timema*. First, the authors examine the progression of chromosome synapsis using SMC3 (a cohesin) and DSB formation and repair, using H2Ax and RAD51, showing that DSBs are formed prior to synapsis. In the second vignette of the manuscript, the authors examine MSCI in *Timema* species, proposing an interesting and supported hypothesis that unsynapsed chromosomes are not sufficient to explain MSCI. In the third section of the manuscript, the authors expand on a recent study from their group characterizing CenH3 during spermatogenesis. The authors then examine centromere dynamics through Bub1, Hdc80, and CenpC analysis, showing that the recruitment of centromere proteins is uncoupled.

*Author response: We thank the additional reviewer for the time and effort invested to review our work.*

The strength of the manuscript is its thorough cytological analysis of various chromosomal events throughout spermatogenesis in a noncanonical model organism. The cytological images are often quite stunning, and I appreciate the time and technical skills meiotic spreads require, particularly in a non-model organism.

Overall, though, the manuscript has various critical flaws. First, the main figures characterize *Timema califoricum*, but the supplemental figures from which the authors drive conclusions have a considerable variation of *Timeama* species and spermatogenesis stages. For instance, *T. cristinae* is analyzed for H2Ax and H3K9me3, but not RAD51, though all three were analyzed in various stages in *T. petita* and *T. chumah.* None of these were analyzed for CenH3 or NDC80, as *T. knulli* and *T.* douglasi were, however in random stages. As a result of this wide inconsistent variation of analyses, it is unknown and arguable about how conserved some of these events truly are within the *Timema* species.

*Author response: We understand the reviewer’s concern. However, the inconsistency across species and stages is primarily caused by the limited number of meiotic cells at various stages and the limited number of specimens per species we can obtain from this group of non-model organisms. To be transparent on the above limitations, we added a section “Limitations of the study” in Material and methods. In this section, we notably emphasize that our conclusions on each cellular process were drawn by comparing Timema species that represent different clades in the phylogeny. For clarity, we also added a supplementary table “Supplementary table 3”, where the number of individuals and technical replicates used for each species and antibody is reported.*

Second, the format of the manuscript was very difficult to follow. Content and information that are typically in an introduction are in the results, while results are often in the Figure Legends. Further, the results within the manuscript read as separate, disparate studies, as opposed to one comprehensive study. Due to the format, the manuscript was extremely difficult to follow and impedes the ability to ascertain the larger significance of the study. To address this concern, the authors can reformat the manuscript, ensuring that it is more readable to your audience.

*Author response: We have made substantial revisions regarding the manuscript’s formatting and clarity to address this concern. We provide a rewritten manuscript with clearly separated introduction, results, and discussion sections, as well as better cohesion between the different cellular processes described to facilitate the reading into a more comprehensive study.*

Other than those major concerns, which focus on manuscript and narrative cohesion, below are additional comments.

**Additional concerns**

1. The authors do not reference the letter of the panel as they describe results in the figure (e.g., Fig 1A, etc.).

*Author response: this is fixed.*

2. The supplemental figures are barely mentioned in the text, if at all, though they contain much data. As a result, they are distracting and I am unsure at where and what panel I should be looking.

*Author response: we now more explicitly reference to the supplementary figures and to the different panels they contain.*

3. In most figures - Spermatid row: These results aren’t mentioned in the text. Are they interesting? What is important here?

*Author response: we now included, along the text, descriptions and interpretations of the results for each staining on spermatids.*

4. Sup Fig 3 – The text says chiasmata across 5 *Timema* species, but there are only three.

*Author response: this sentence was replaced by “(A’-L’) illustrate rod and ring bivalents across 4 Timema species at metaphase I.”*

5. Authors mis-reference published articles (e.g., “Toubiana and Jeli’s papers” and “(ref)” throughout the manuscript

*Author response: this is fixed.*

6. Typo: second paragraph of results and discussion – “Double strand break” should be “double strand break”

*Author response: this is fixed.*

7. The authors introduce RAD51 as a “DNA repair protein”. It is important that they mention that it is a marker of “early” DNA repair, as just a general or a later DNA repair protein would lend a different interpretation than what the authors provide.

*Author response: We changed this sentence by “Meiotic recombination is initiated by the formation of programmed DNA double-stranded breaks (DSBs) that rapidly induce the phosphorylation of the histone variant H2AX (γH2AX) and the recruitment of the recombinase Rad51 that is involved in homologous recombination and mediates the single-strand invasion to repair DNA after DSBs.”, which specifies the “early” action of Rad51 in DNA repair.*

8. Figure 1/2 – The authors say “late leptotene/early zygotene” but do not have it labeled as such on the Figure.

*Author response: we changed “late leptotene/early zygotene” by “early zygotene”.*

9. Figure 1 - The H2Ax foci is difficult to see, as the signal is dim in the images.

*Author response: we adjusted brightness and contrast to improve the brightness of the signal and added zoomed insets.*

10. Supplemental Figure 5 – magenta stain says “Rad51” but should be “CenH3”

*Author response: this is fixed.*

11. Figure 5 schematic should be moved to supplemental, as it summarizes findings from a previous study.

*Author response: this is now the Supplementary figure 5.*

12. Figure 8 – Ndc80 staining in Pachytene, Diplotene, and Meta is difficult to see.

*Author response: no signal is observed for Ndc80 in Pachytene and Diplotene. We refer to these results in lines 314-318. We adjusted brightness and contrast for the Metaphase I acquisition to improve the signal.*

13. Bub1 staining in Supplemental Figure 9 shows much background, and as a result, it is difficult for me to interpret.

*Author response: we zoomed and adjusted the contrast and brightness as much as possible to highlight the foci on each chromosome.*

14. A comprehensive schematic of the findings at the end of the manuscript would help tie all the results together.

*Author response: the main findings of our study are summarized in Supplementary figure 5.*

Comments to the editor:

I truly believe this manuscript has the potential to provide novel insights into spermatogenesis in a non-model organism, which is critically needed when considering the importance of evolutionary processes. However, in its current form, this manuscript is not there, simply because of the confusing format and the writing. I highly suggest that the authors provide a complete re-write but with the same data, focusing on making the manuscript more cohesive and to read as a comprehensive study (as opposed to multiple disparate studies).

REFERENCES

1.	Djordjevic J, Van PT, Toubiana W, Labédan M, Dumas Z, Aury JM, et al. Dynamics of X chromosome hyper-expression and inactivation in male tissues during stick insect development. Plos Genet. 2025;21(3).

2.	Toubiana W, Dumas Z, Van PT, Parker DJ, Mérel V, Schubert V, et al. Functional monocentricity with holocentric characteristics and chromosome-specific centromeres in a stick insect. Sci Adv. 2025;11(1).

3.	Jagannathan M, Cummings R, Yamashita YM. A conserved function for pericentromeric satellite DNA. Elife. 2018;7.

4.	Dernburg AF, McDonald K, Moulder G, Barstead R, Dresser M, Villeneuve AM. Meiotic recombination in C. elegans initiates by a conserved mechanism and is dispensable for homologous chromosome synapsis. Cell. 1998;94(3):387-98.

5.	Viera A, Santos JL, Page J, Parra MT, Calvente A, Cifuentes M, et al. DNA double-strand breaks, recombination and synapsis:: the timing of meiosis differs in grasshoppers and flies. Embo Rep. 2004;5(4):385-91.

6.	Page J, de la Fuente R, Manterola M, Parra MT, Viera A, Berríos S, et al. Inactivation or non-reactivation: what accounts better for the silence of sex chromosomes during mammalian male meiosis? Chromosoma. 2012;121(3):307-26.
