# Supplementary material for: Dynamics of recombination, X inactivation and centromere proteins during stick insect spermatogenesis
Source: PLoS Genet. 2025 Aug 18;21(8):e1011827. doi: 10.1371/journal.pgen.1011827 (PMC12393702; doi:10.1371/journal.pgen.1011827)
Supplement: Attachment — Submitted filename: Response to Reviews.docx [file pgen.1011827.s005.docx]

*General author’s response: we thank both reviewers for their constructive comments and would like to especially thank the first reviewer for taking the time to reevaluate our work. The manuscript was revised in order to address the different comments that were raised (see the point-by-point responses below).*

Point-by point response to reviews

**Reviewer: 1**

The authors have addressed most of my concerns, and the new version of the text is more

streamlined and easier to read compared to the original version. I feel that once the

following minor comments are addressed, the manuscript is suitable for publication in

PLOS Genetics.

Minor comments:

- Authors need to be consistent with recombination wording when referring to Rad51.

While the authors are correct in their rebuttal that Rad51 is often used as a marker

of recombination, it marks recombination INITIATION, and stating “recombination

happens before synapsis” makes it sound like recombination is COMPLETE before

synapsis, which is impossible. The authors should consistently say “recombination

initiation”, when referring to Rad51 foci.

- Ex - Line 122 of p32, “homologous recombination” à “INITIATION of

homologous recombination”

- Ex - Line 364 of p42: Change “recombination” à “recombination INITIATION”

*Author response: We replaced “homologous recombination” by “the initiation of homologous recombination” when referring to Rad51 throughout the text.*

- Antibody staining in figures is still too weak and hard to see, especially in the

merged images. All channels need to be made brighter, and it would be very helpful

for the reader to show 3-way merges between all channels in all figures.

*Author response: we increased the brightness of the figures where the signal was difficult to see, including the figures illustrating γH2AX signals that were pointed out by the two reviewers. However, we did not include a three-channel merge, as it resulted in overly cluttered images that were difficult to interpret.*

- Line 72-73 of p30: Change “In most eukaryotes,” to “In many eukaryotes,”

Holocentricism is probably under-appreciated in plants and insects, which greatly

outnumber vertebrates, due to the low number of species actually studied.

*Author response: this is done.*

- Line 86 p30, “they also pose significant challenges for preserving genome integrity”:

Please describe how and what kind of challenges.

*Author response: we precise that these challenges are notably in regards to DNA breaks in haploid cells.*

- Incorrect referenced figures:

- Line 146 of p33, “SMC3 filaments become interrupted near chromosomal

ends”: This is only clearly shown in Supplementary Figure 3A’-O’ with the

inserts. Other figures should be not referenced here, or it needs the same

inserts as in Supp Fig 3.

*Author response: we agree and now only use Supplementary figure 3 as reference.*

- Line 308-309 of p39-40: Leptotene and zygotene stages are referred here.

Only Figure 6A-J, Figure 7A-E, Figure 8A-E should be referenced here.

*Author response: indeed, we modified the text accordingly.*

- Line 152-153 of p33, “two Timena species forming one “ring” bivalent”: Do these

two species ALWAYS have one ring bivalent? How often was the ring bivalents

observed?

*Author response: We did not quantify the exact frequency of cells with or without ring bivalents. Cells without ring bivalents (i.e., with a single chiasmata) do exist but are clearly a minority. We made this clearer in lines 170-173.*

- Line 163 of p33, “evidence”: Antibody validation concerns. While I am not

concerned about the SMC3 antibody (chromosome axis in meiosis has an

unmistakable structure), the foci of H2AX and Rad51 are less convincing. The Rad51

seems more real than the H2AX signals, as the signal seems to be a bit more

specific, but the authors really need to include a western blot or some other kind of

validation for these antibodies, such as Bleomycin test that will show more of both

H2AX and Rad51 after bleomycin treatment if that’s real signal. I do not see any

validation of the antibodies, even in the other papers mentioned.

*Author response: We explicitly acknowledge in the manuscript that “because we used a commercial antibody against γH2AX, we cannot formally exclude that this variation across species is caused by variable antibody affinities or cross-reaction with other proteins” (lines 206-208).* *That said, we note that the γH2AX and Rad51 antibodies used in our study are the same commercial products previously validated in grasshoppers, and reveal a similar and expected pattern at a polarized region of the nucleus during zygotene across all Timema species and individuals.*

*As recommended by the reviewer, antibodies against γH2AX and Rad51 proteins were tested by western blot using protein extracts from T. cristinae. This revealed a band at the expected size (i.e., around 15 kDa) for the γH2AX antibody, while no band could be detected for the Rad51 antibody. This information is now reported in the “Design, selection, and validation of antibodies” section of the Material and methods (lines 503-506). The suggested treatment with bleomycin is not possible in Timema due to our difficulty to raise them in lab conditions.*

- Line 182 of p34, “compared to both insects and mammals”: Timena is an insect too.

Change to “compared to other insects and mammals”

*Author response: this is fixed.*

- Despite the characterization of spermatogenic stages in the methods, it is still not

clear to me how leptotene and zygotene are distinguished here… for example - Line

206-207 of p35, “(i.e., leptotene and zygotene; Figure 3A-j)”: To me, these look

basically the same.

*Author response: We agree that distinguishing leptotene from zygotene can be challenging. In our study, we define the zygotene stage as the transitional phase beginning with the appearance of the bouquet configuration (here observed in Figure 3F with the telomere clustering) and continuing until the formation of thick SMC3 filaments, which marks the progression of synapsis. Accordingly, we define leptotene as the stage characterized by the presence of only thin SMC3 filaments and the absence of telomere clustering.*

*To clarify this distinction, we have revised the relevant section of the text (lines 574–578) to explicitly state the criteria used to differentiate these two stages.*

- Line 211 of p35, “(Figure 3a-e’, etc.)”: Confusion about the two cells in a vs a’. Why

are two diferent cells shown and why does only one cell have an “X body”?

*Author response: the two cells correspond to the X-containing and nullo-X round spermatids (as typical for species with XX/XO sex determination), which provides the evidence that the X is the exclusive chromosome not transcribed at this stage. Those two types of cells are illustrated and described in the Supplementary figure 3.*

- Some text in the results section need to be moved to the discussion:

- Line 227-262 of p36-38

- Line 333-335 of p41

- Line 340-350 of p41, “Initially, CenH3….”

- Line 351-356 of p41

*Author response: Reviewer#2 made a similar suggestion but as stated below, we believe that the sections in question provide a necessary summary of the cellular dynamics observed across multiple proteins, and overall help integrating and contextualizing the findings. Furthermore, we prefer to center the Discussion on the various hypotheses concerning the origin and dynamics of these processes, and on how our findings provide insights into resolving them.*

*Note, however, that we relocated the portion from line 351 to 356 to the Discussion section.*

- Line 290 of p39: Change “all Timena species” à “all EXAMNIED Timena species”

*Author response: this is changed as suggested by the reviewer.*

- Line 366-368 of p42, “… but absent in only D. melanogaster and C. elegans. It may

also reflect the ancestral state in insects. Indeed, only in female meiosis of

Drosophila (Diptera order) is synapsis known to precede recombination.”: These

sentences are unclear and seemingly redundant.

*Author response: for clarity, we removed the sentence “It may also reflect the ancestral state in insects.” and modified the following sentence by “In insects, only Drosophila female meiosis (Diptera order) is known to have synapsis preceding recombination.”*

- Line 370-372 of p42, “Bombyx mori”: There still seems to be a lack of understanding

regarding recombination initiation and completion. Recombination nodules

observed in B. mori by EM after synapsis indicate the recombination doesn’t

COMPLETE until after synapsis. There is currently no data on recombination

initiation and pairing or synapsis in Bombyx or other moths.

*Author response: as recombination nodules occur as early as the beginning of the zygotene stage and before the completion of synapsis along the entire length of all chromosomes, we interpreted these results as evidence for recombination initiation before the completion of synapsis. But we acknowledge that gradual synapsis on small sections of the chromosomes directly followed by recombination cannot be excluded. We therefore removed this reference from the revised manuscript.*

- Line 428-429 of p43-44: Senaratne et al. (2021) should be referenced here.

*Author response: in this sentence we refer specifically to the possible mechanisms involved in CenH3 deposition. Work from Senaratne et al. (2021) describe a species in which CenH3 is absent and thus not appropriate as reference here.*

- Supp Fig 4: Arrows marking X chromosome should be included in all panels, not just

DAPI panel

- Supp Fig 4, line 687 of p61: Missing/mislabel “H, I, J”

- Supp Fig 4, line 687 of p61: Missing description of “SMC3” in figure legend

*Author response: Supplementary figure 4 was modified accordingly and a description for the absence of SMC3 in round spermatids was added.*

- Typos:

- Line 159 of p33: Typo in “recombination-dependent machinery” à

“recombination machinery-dependent”

*Author response: we kept our formulation since we want to refer to a machinery that depends on recombination (we are not referring to the recombination machinery here).*

- Line 183 of p34: Typo in “as” à “at”

*Author response: done*

- Line 441 of p45: Typo in “Cenh3” à “CenH3”

*Author response: done*

- Line 635 of p55: Typo in “(a-o)” à “(a-j)”

*Author response: done*

Reviewer #2: The manuscript submitted by Dumas and coworkers is an interesting study, characterizing three different aspects of meiosis in several species of stick insects belonging to genus Timema: dynamics of recombination and synapsis, inactivation of the X chromosome, and loading of centromeric proteins. The authors performed the immunolocalization of different proteins involved in these processes (SMC3, γH2AX, RAD51, H3K9me3, RNA pol-II, CenH3, Cenp-C and Bub1). Overall, the results indicate that stick insect species follow a meiotic pattern, regarding the sequence of recombination-synapsis, similar to other insects like grasshoppers and bugs. In addition, they found some differences in the dynamics of X chromosome inactivation. Finally, the most interesting and novel contribution is related to the location and dynamics of centromeric proteins, a topic that has been particularly elusive in the study of meiosis in insect.

Although the work is mainly descriptive, the comparison between different species and the inclusion of centromere analysis make it a relevant contribution. Nevertheless, there are some aspects that should be addressed by the authors (this is not an exhaustive list).

General comments:

1- the work is mainly focused on the species T. californicum. Nevertheless, the same detailed analyses are presented for almost every protein in the other species. I am not sure about why authors choose this option, but this may bias the interpretation and discussion of the results towards the patterns found in this species. It would be better if this discussion was set up from an unbiased perspective.

*Author response: the aim of this manuscript is twofold: 1) to provide a detailed characterization of spermatogenetic process in Timema, 2)* *to highlight the diversity observed both within the genus and in comparison to other taxa.* *To achieve this and ensure clarity, we begin by focusing on a single reference species, T. californicum, before examining patterns of conservation and divergence across other Timema species where data are available.*

2- the morphological description of meiosis is too lengthy in many parts of the text. Likewise, some parts of the text seem superfluous or not properly located along the manuscript. Particularly, sections from line 227 to 262 and from line 337 to 356 could be completely eliminated or partially incorporated into the discussion section.

*Author response: We appreciate the reviewer’s suggestion and understand the concern regarding the length and placement of the morphological description. However, we believe that the sections in question provide a necessary summary of the cellular dynamics observed across multiple proteins, which is essential for a full understanding of the processes under study. For this reason, we feel it is more appropriate to retain these summaries at the end of the Results section, where they serve to integrate and contextualize the findings.*

*That said, in response to the reviewer’s suggestion, we have revised the structure slightly by relocating the portion from lines 351 to 356 to the Discussion section.*

3- In all figures, but specially in those showing figures γH2AX, illumination of the magenta channel is too dark. I could hardly see the signals that authors describe in the text. I suggest carefully revising the levels of the images to make the signals clearly visible.

*Author response: as explained in response to reviewer 1, we modified the brightness of several figures, including the figures illustrating γH2AX signals for better visibility.*

Specific comments:

4- line 68: I would better say: “Following prophase-I events, two rounds of cell…..”

*Author response: we modified the sentence as suggested by the reviewer.*

5- line 86: “ Despite…..” I suggest eliminating this sentence.

*Author response: we think it is important to keep this sentence and state that we still know very little about the processes underlying spermiogenesis and its diversity across species. For example, the diversity in transcriptional activation that appears to occur during spermiogenesis could be very informative for the role of MSCI.*

6- line 120: “…recruitment of the recombinase RAD51…” I would add DMC1 as well.

*Author response: DMC1 is well studied in mammals but remains poorly characterized in other organisms such as insects. We thus prefer referring to Rad51 only, as it has been described in a broader range of organisms, especially since our study does not provide any information on DMC1.*

7- line 160: “… formation as observed in Arabidopsis, yeast, jellyfish and mouse (35-38).” Grasshoppers and bugs, previously cited by the authors, must be added to this list.

*Author response: we specifically reference studies on these organisms because they provide functional evidence (e.g., knock-out experiments) supporting a recombination-dependent machinery for synapsis formation. In contrast, only associations based on temporal relationships can be made in grasshoppers and true bugs.*

8- line 272 and following. The point regarding the loading of CenH3 and the transcriptional activity is too confusing to me. It is not clear why authors try to make a relationship between both processes. This is perhaps related to the fact that in some species the loading of CENP-A is driven by the transcription of repetitive sequences at the centromeres. However, the discussion about this topic seems quite artificial to me. Indeed, the correlation claimed by the authors about the increase of transcriptional activity and the loading of CenH3 might be just a temporal coincidence, and not a correlation at all. Moreover, CENP-A is usually loaded at G1 stage of the cell cycle. Thus, I recommend reassessing this part of the discussion. If authors want to make a point about the relationship between meiosis progression and loading of centromeric proteins, they should revise some of the works by Suja and coworkers (PMID: 19283064, 12584241, 19283064, among others).

*Author response: we agree that this part of the analysis warrants further explanation in the result section and we have expanded the relevant section §(lines 297–300). Given the unusual dynamics of CenH3 deposition during prophase I —specifically, our observations that CenH3 appears as discrete foci in pre-meiotic diploid germ cells but transitions to a longitudinal distribution during leptotene (see Figure 5 and Supplementary figure 6)) — we started to investigate potential mechanisms underlying this pattern. One potential mechanism stems from the known functional relationships between transcriptional activity and centromere protein deposition in Caenorhabditis elegans and Bombyx mori (citations 45 and 46 in the text). In these organisms, CenH3 and other centromere proteins are specifically deposited in transcriptionally silent regions.*

*We fully agree that the observed timing of CenH3—loading during leptotene when transcription is absent, and reorganizing into single foci when transcription is active—reflects a temporal correlation and does not imply a causal relationship. We explicitly state that our findings support a mechanism based on SMC proteins, we never suggest that we demonstrate such a mechanism. Furthermore, our observations allow us to exclude other proposed mechanisms based on the order of events. Technical limitations in non-model organisms such as Timema currently constrain functional assays to directly assess potential mechanisms.*

*That said, among the three mechanistic hypotheses we examined (i.e., transcriptional activity, kinetochore proteins and SMC proteins) the most plausible explanation for our findings is indeed a functional relationship between CenH3 dynamics and SMC (see Discussion, lines 440-465).*

9- Figures are not completely comparable to each other in terms of the meiotic stages included. Some incorporate different stages of the second meiotic division, but it seems not to be a clear pattern as to why some stages are absent.

*Author response: we dedicated a section in the Material and methods (“Limitations of the study”) to explicitly explain the reasons for this variation, which are inherent to the insect system under study. For example, the lack of cell stages at the second meiotic division is primarily caused by the very few numbers of cells detected at this stage in male gonads (the majority of cells are in pachytene). The limited access to adult males, which are collected from natural populations and can only be found during a short season in the year, further accentuates these constrains, which prevent the consistent description of all stages in all species for a given protein.*

10- line 377 and following. The first sentence of this paragraph does not make any sense to me. Moreover, the following discussion about the origin of MSCI seems a bit rambling and unsupported. The fact that MSCI does not protect sex chromosomes from the production of DSBs is quite obvious from what we know in mammalian models. On the other hand, the need for silence a few genes in the X chromosome is a mere speculation in these species and is not a completely satisfactory explanation as to why the X chromosome is completely inactivated. I suggest revising the work by Viera et al., (PMID: 39766780, 34946793) for additional explanations of X chromosome inactivation in insects.

*Author response: In mammals, MSCI is thought to represent a general response to unsynapsed chromosomal regions (MSUC – meiotic silencing of unsynapsed chromatin). However, whether this is also the case in groups such as insects is not known, and a broader consideration of hypotheses, even if they have been excluded in mammals, is therefore relevant. To improve clarity for readers working on mammals, we have revised the sentence as follows: “We also reveal that the lack of synapsis on the Timema X chromosome during the male pachytene stage is associated with the initiation of MSCI, as shown in several organisms, including mammal and other insect species.” However, this hypothesis is not sufficient to explain that the X chromosome remains inactivated well after synapsis is presumed complete. Furthermore, the reasons behind the evolution and temporal dynamics of MSCI are still debated and may vary among taxa. In support of this complexity, a study from Viera et al., shows that MSCI coincides with γH2AX signals prior to pachytene in grasshopper spermatogenesis, suggesting a role in DSB protection. We show this potential role is not supported in Timema.*

*Regarding the possible link between transcription and MSCI; in Timema, MSCI initiates at pachytene—precisely when autosomal transcription becomes active—and resolves during the round spermatid stage, when autosomal transcription ceases. Supporting also a link between transcription activity and MSCI establishment, in grasshoppers—where autosomal transcription begins earlier, during leptotene—Viera et al. observed the formation of a sex body at that stage, which dissipates during spermiogenesis, coinciding with the end of transcription.*

*Nonetheless, we acknowledge that this proposed link between transcription and MSCI remains speculative in the absence of functional validation. Accordingly, we have presented it as a working hypothesis in the Discussion section.*

11- Discussing the configuration of centromeric proteins in holocentric chromosomes is interesting, but monocentric chromosomes of Timema species may not fit a similar pattern.

*Author response: in this part of the discussion, we only refer to holocentric chromosomes as their centromere proteins are deposited along the chromosomal axis during early meiosis, similarly to the CenH3 protein in Timema. These similarities, led us to suggest a functional relationship between centromere and SMC proteins, as modeled recently to explain the assembly of centromeric nucleosomes in holocentric species (citation 61 in the manuscript).*

12- I believe that Rocío Gómez Ortega is incorrectly acknowledged. I suspect authors may refer to Rocío Gómez Lencero, from Universidad Autonoma of Madrid. Please, correct.

*Author response: Indeed, and we apologize for this mistake… this is now corrected.*
